# A Review of Recombination Coefficients of Neutral Oxygen Atoms for Various Materials

**DOI:** 10.3390/ma16051774

**Published:** 2023-02-21

**Authors:** Domen Paul, Miran Mozetic, Rok Zaplotnik, Gregor Primc, Denis Đonlagić, Alenka Vesel

**Affiliations:** 1 Jozef Stefan Institute, Jamova cesta 39, 1000 Ljubljana, Slovenia; 2 Jozef Stefan International Postgraduate School, Jamova cesta 39, 1000 Ljubljana, Slovenia; 3 Faculty of Electrical Engineering and Computer Science, University of Maribor, Koroska cesta 46, 2000 Maribor, Slovenia

**Keywords:** heterogeneous surface recombination, recombination coefficient, surface catalicity, catalytic efficiency, atom loss coefficient, oxygen, neutral atoms, plasma

## Abstract

Relevant data on heterogeneous surface recombination of neutral oxygen atoms available in the scientific literature are reviewed and discussed for various materials. The coefficients are determined by placing the samples either in non-equilibrium oxygen plasma or its afterglow. The experimental methods used to determine the coefficients are examined and categorized into calorimetry, actinometry, NO titration, laser-induced fluorescence, and various other methods and their combinations. Some numerical models for recombination coefficient determination are also examined. Correlations are drawn between the experimental parameters and the reported coefficients. Different materials are examined and categorized according to reported recombination coefficients into catalytic, semi-catalytic, and inert materials. Measurements from the literature of the recombination coefficients for some materials are compiled and compared, along with the possible system pressure and material surface temperature dependence of the materials’ recombination coefficient. A large scattering of results reported by different authors is discussed, and possible explanations are provided.

## 1. Introduction

For several decades, oxygen plasma has seen widespread use in various industrial applications, ranging from advances in the food industry [1,2,3] to uses in the bustling semiconductor industry [4,5,6], novel approaches in medicine [7,8,9], and various other applications [10]. The use of plasma is even more prevalent in many fields of research, from nuclear fusion [11,12], studies on cell growth [13], and various advances in surface science [14], such as surface modification [15,16], surface functionalization [17], sterilization [18], etc.

Such widespread use of plasma demands research into the characterization of the plasma itself. Many researchers have characterized different properties of different plasmas sustained by various discharges, from neutral atom density [19], ion density [20], electron density [21], and energies of plasma particles [22], to the plasma emission spectra in the optical [23], infrared [24], and ultraviolet ranges [25]. Such studies are very important in understanding plasma discharges, but the interactions of plasma with different materials must not be overlooked to devise any applications from such studies.

Interactions of plasma with the surface of a material are a complex topic, with many different plasma particles interacting with the surface in different ways. On the one hand, plasma discharges emit radiation in a broad range from infrared (IR) to vacuum ultraviolet (VUV), which can strongly interact with certain surfaces and has been studied in ref. [26]. The plasma particles, such as positive and negative ions, free electrons, excited neutral molecules, and radicals, can also interact with the surface. High-energy ions can have the most drastic effects on a surface, from etching [27] to ion implantation [28,29], while free electrons have hardly any effects on a surface and are more commonly utilized in different measurement methods [30]. Setting aside charged particles, we will focus on the neutral particles. Among those are rotationally or vibrationally excited molecules [31] and, more important for this paper, molecular radicals. Since oxygen plasmas are most often used, our attention will be focused on the interactions of oxygen plasmas with surfaces, in particular, on the interaction of neutral oxygen atoms. To be specific, the major reactants in many oxygen plasmas suitable for tailoring the surface properties of solid materials are, in many cases, neutral oxygen atoms in the ground state [32].

Neutral oxygen atoms are mainly created in the plasma discharge, where parent oxygen molecules are dissociated at a collision with an energetic electron. In some cases, neutral atoms can also be created from molecules dissociating on the material surface via heterogeneous surface atomization [33]. Our interest is in the reverse process, the heterogeneous surface recombination of neutral oxygen atoms.

There are two widely accepted mechanisms describing neutral atom recombination. The first is the Langmuir–Hinshelwood mechanism [33], which describes the recombination of two neutral oxygen adatoms (O(s)) after they adsorb to the surface. Once they diffuse on the surface (S):

(1) $$S+O\left(s\right)+O\left(s\right)\to O_{2}+S.\;$$

We obtain the resulting oxygen molecule ($O_{2}$), which can leave the surface through desorption [33]. Such molecules are in thermal equilibrium with the surface, and their energy depends on the temperature of the surface. The second mechanism is the Eley–Rideal mechanism [33], which describes an adatom at the surface recombining with an incident atom from the gas (O(g)):

(2) $$S+O\left(s\right)+O\left(g\right)\to O_{2}+S.\;$$

The resulting molecule is not necessarily in thermal equilibrium with the surface due to the molecule receiving energy from the incident atom [33].

Regardless of the mechanism of neutral atom recombination, we can define the recombination coefficient (γ) of a certain surface as the ratio between the flux of incident neutral atoms ($j_{atoms}$) and the number of recombined molecules leaving the surface in a unit of time ($j_{molecules}$):

(3) $$\gamma =\frac{2\;j_{molecules}}{j_{atoms}},$$

with factor 2 to counterbalance the fact that two atoms create a single molecule. Since not every atom which reaches the surface recombines with another atom into a molecule, and on the opposite side, there is always an (even infinitesimally) small fraction of atoms that do recombine at the surface, the recombination coefficient has values between 0 and 1.

Often, the term «recombination coefficient» is used interchangeably with the terms catalytic efficiency, surface catalicity, and sometimes with the term atom loss coefficient. The latter also takes into account any other losses of neutral atoms at the surface, including physisorption, chemisorption, implantation, etc. [33]. While it is perfectly valid to consider such processes, we will be forgoing that. The first reason is that surface recombination is usually the prevalent process in plasma–surface interactions [33,34]. The second reason is that we are dealing with oxygen plasma, where most samples have had their surface already exposed to oxygen prior to any measurements. Thus, an oxide layer already appeared prior to exposure to oxygen plasma, minimizing any atom losses due to oxidation-related processes. Therefore, all further mentioning of recombination coefficients will not distinguish between actual surface recombination and combined surface atom losses.

This paper will examine scientific articles by different authors and their methods for determining the recombination coefficients of oxygen atoms on surfaces of various solid materials. Firstly, we will briefly go through various plasma systems and gaseous discharges, as well as the choice of parameters for said discharges, focusing mainly on the total pressure of gas in the experimental system and the surface temperature of the examined materials. We will also compare the resulting recombination coefficients achieved in similar experimental setups and explain any discrepancies. Along with direct current (DC), radiofrequency (RF), and microwave (MW) discharges, some theoretical models for determining the recombination coefficient will be examined.

Secondly, we will describe different measurement techniques for determining the recombination coefficient, such as the widely used actinometry, two-photon absorption laser-induced fluorescence (TALIF), calorimetry, etc. We will categorize the techniques for easier comparison of determined recombination coefficients.

Lastly, we will compare the recombination coefficients of different materials and divide the materials into three groups: catalytic, semi-catalytic, and inert materials, depending on the materials’ recombination coefficient.

The motivation for such a review article is to provide readers easier access to somewhat comprehensive literature on recombination coefficients of neutral oxygen atoms on the surfaces of different materials, which is important information that needs to be known either when designing experiments or when interpreting and discussing results. Additionally, since recombination coefficients may depend on system pressure and surface temperature, we pay special attention to reported pressures and temperatures and their effects on the recombination coefficient. Less comprehensive lists have been compiled in other review articles, namely for neutral nitrogen atoms [35], modeling nitrogen–oxygen hybrid systems [36], general atom recombinations on surfaces [37], recombination coefficients determined via the spinning wall method [38], a study of atom recombinations on high-temperature materials [39], and a study of the pressure dependency of the recombination coefficient in a shock tube system [40].

## 2. Low-Pressure Discharges as Sources of Oxygen Atoms

The type of gaseous discharge used for ignition and sustaining gaseous plasma can alter the interaction of plasma with a surface due to varying densities and energies of plasma particles. Therefore, different low-pressure discharges will be presented in this section, starting with the more common and ending with more exotic and less frequently used discharges, as well as theoretical models. Non-equilibrium gaseous plasma can also be sustained at atmospheric pressure, but the loss of atoms at elevated pressures is predominantly in the gas phase at three-body collisions, so atmospheric pressure plasmas are not feasible for studying the surface recombination phenomenon.

### 2.1. DC Discharges

**Figure 1:**
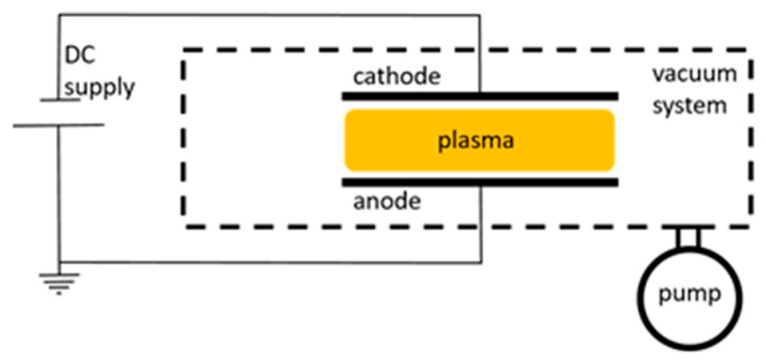
Schematic of a typical DC plasma system.

The simplest discharge is a DC discharge, schematically shown in Figure 1. A DC plasma discharge can be created by applying a sufficient voltage of several hundred volts to a volume of a gas at low pressure (e.g., below 7000 Pa). As a primary electron is released from the cathode and accelerated by the applied electric field, it can ionize atoms in the volume of gas, which also produces electrons, which, in turn, become accelerated [41]. Thus, a sustainable plasma discharge is created.

In the proceeding articles, different types of DC discharges have been used, from regular DC glow discharges used by Lopaev et al. [42,43] to the pulsed DC discharges used by Cartry et al. [44,45] and to variations of an arc discharge used in NASA’s arc jet facilities [46]. The DC glow discharges operate in the normal glow mode with lower applied voltages. In such a mode, the surface area of the cathode, covered in luminous plasma, is proportional to the current [41]. In a pulsed DC discharge, the same glow discharge is continuously switched on and off. With the correct timing of these pulses, the neutral atoms remain in the system in between the pulses, while other species (ions, free electrons) are mostly eliminated [47]. This provides plasma with a higher concentration of neutral atoms with respect to the time-averaged concentrations of charged particles compared to regular DC glow discharges. As for arc jet discharges, they are a more exotic approach to a DC discharge, where the high discharge current creates an unstable discharge with arcs forming between electrodes [41].

### 2.2. RF Discharges

RF discharges are the most prevalent both in academic studies and in industry. During the process of collecting data for this article, around two-thirds of all data found was produced in an RF discharge system. Unlike DC discharges, the current (and the electric field) alternates in an RF discharge. The frequencies of such discharges are usually set at 13.56 MHz. The RF voltage generates an electric field that oscillates, thus accelerating electrons. Like in the DC discharge, this results in electrons with sufficient energy triggering ionizations. Among some of the advantages of using an RF discharge over a DC discharge is a more efficient ionization, resulting in a plasma with a denser plasma particle population [48].

Three different RF discharges can be distinguished: capacitively coupled plasma (CCP), inductively coupled plasma (ICP), and pulsed RF discharge [34]. In CCP discharge, the plasma is capacitively coupled to an RF generator. A sheath is formed between the electrodes and bulk plasma, and it oscillates with the same frequency, albeit not in phase with the electrodes [41]. Unlike its widespread use in various industries, the use of CCP discharges was rare in the reviewed literature concerning recombination coefficient determination. Some combined use with ICP discharges was reported by Mozetič et al. [49] and Gomez et al. [50], but the exclusive use of CCP discharges seems to be limited to Tsutsumi et al. [51], Tserepi et al. [52], Shibata et al. [53], and Rakhimova et al. [54].

**Figure 2:**
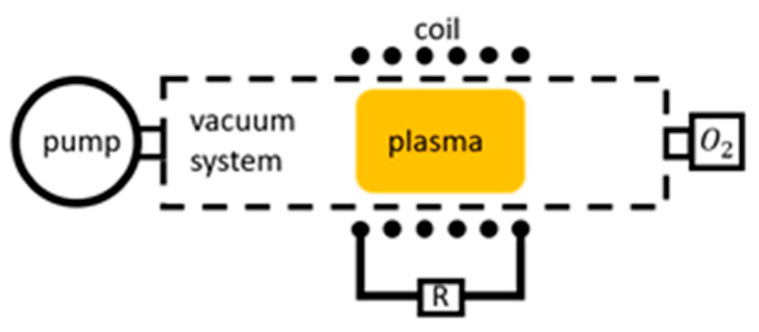
Schematic of a typical inductively coupled radiofrequency plasma discharge.

The amount of experimental results using CCP discharges is overshadowed by the use of ICP discharges, which represent around half of all results presented in this paper. The ICP discharge is created by inductive coupling between an RF transmitter and the ionized gas in the system. The transmitter can be a simple antenna or a coil around the plasma reactor, as shown in the example in Figure 2. The electrons receive their energy via the oscillating electromagnetic field. The advantage of an ICP discharge over a CCP discharge is the ability to achieve higher plasma densities [41]. ICP discharges were used in experiments determining catalytic coefficients in seminal works by Melin and Madix [55], Graves and Linnett [56], Dickens and Sutcliffe [57], and Linnet and Marsden [58]. ICP discharges remain popular to this day, seeing use in works by Mozetič et al. [59], Wickramanayaka et al. [60], Stafford et al. [61], and many others.

RF discharges may also operate in the pulsed mode. Here, RF power is applied in pulses that may vary in length and frequency. One of the compelling reasons to choose a pulsed RF discharge over a static one is to avoid heating the plasma system with bulk plasma, which can negatively impact the measurements. Authors such as Matsushita et al. [62], Myerson [63,64], and Guyon et al. [65,66] have successfully used a pulsed RF discharge to determine the recombination coefficient of various materials.

### 2.3. MW Discharges

**Figure 3:**
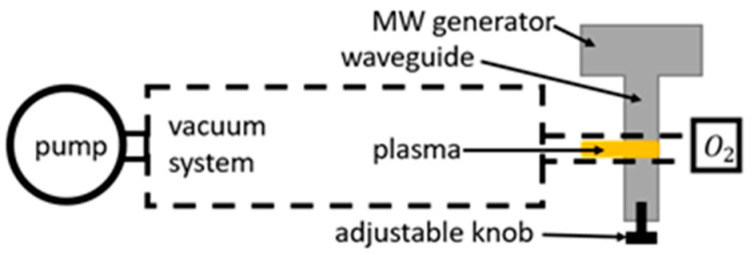
Schematic of a magnetron MW plasma system.

Similarly to RF discharges, MW discharges are ignited and sustained by high-frequency oscillating electromagnetic fields. A standard MW frequency most commonly used in MW discharges is 2.45 GHz, which provides wavelengths comparable to experimental-size plasma reactor dimensions [67]. Because of such high frequencies, practically all the energy is transferred to electrons, triggering ionizations [68]. The same is true for other high-frequency discharges (namely RF). Generally, MW discharges produce high densities of radicals, such as neutral oxygen atoms, compared to other types of discharges. There are different types of MW discharges based on the source of microwaves, such as cyclotron or magnetron (Figure 3).

Most authors experimenting with MW discharge systems did not specify the type of discharge used. A few exceptions include Booth and Sadeghi [69], who used a cyclotron to determine the recombination coefficient of stainless steel. A magnetron was used by Greaves and Linnett [70] in their study of oxygen atom recombination on quartz, as well as by Kaufman when studying quartz [71] and Pyrex [72].

Along with the source of microwaves, discharge requires something connecting the source with the reactor. Waveguides and MW cavities are most commonly used. The MW cavity, which acts as a special resonance chamber to strengthen the electromagnetic field, was used by Hacker et al. [73] in their studies of platinum and quartz as well as by Brake et al. [74,75] in their study of recombination on quartz glass. Zaplotnik et al. [76] reported using a type of MW cavity called a surfatron when studying the recombination coefficients of different polymers. Cartry et al. [77] also reported using a surfatron in their studies of quartz glass. A plasmatron, which is another type of MW cavity, was used by Kolesnikov [78] when studying quartz glass. The use of a waveguide was reported by Balat-Pichelin et al. [79,80,81,82,83,84] in providing a great set of data for a variety of materials. While the rest of the authors did not provide the full details of their method, their studies of recombination coefficients for various materials are nevertheless of great importance. We will examine two additional sources of atoms useful for studying heterogeneous surface recombination.

### 2.4. Other Discharges

**Figure 4:**
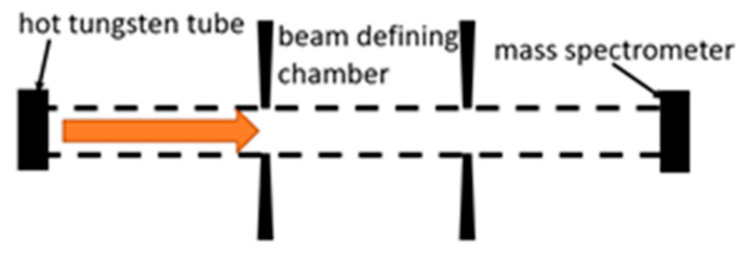
Schematic of the atomic beam plasma system, which was exclusively used in conjunction with mass spectrometry in the referenced works.

In addition to the most common discharges presented above, other setups were also used to investigate recombination coefficients, such as the atomic beam used by Sjolander [85]. In this work, a heated tungsten ribbon (at approximately 3000 K) is exposed to molecular oxygen, which dissociates into atoms. Due to high surface temperature, desorption of atomic oxygen is possible, which is then drifted towards a metal sample (Figure 4).

**Figure 5:**
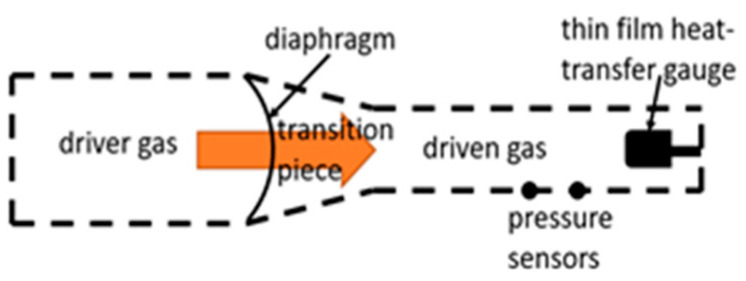
Schematic of a typical shock tube plasma system.

Another type of discharge is the shock tube, used by Yang et al. [40], Park [86], and Goulard [87]. This type of discharge is used to replicate blast waves, which occur during violent explosions. In essence, a shockwave is generated on one side of the tube by the driver gas at a higher pressure, which, upon coming into contact with and rupturing a membrane, pushes the lower-pressure driven gas to the other side of the said membrane (Figure 5). Due to the high energy of the shockwave, plasma may be formed.

### 2.5. Models

Some researchers used simulations instead of experiments to determine the recombination coefficient (γ) of different materials. While their models vary in both approach and scope, some common denominators may be taken from their research. What is most interesting is that for a given material, the proposed surface temperature dependence of the recombination coefficient seems to take the form of:

(4) $$\gamma \;\left(T_{s}\right)=Ae^{-B/T_{s}},$$

where A and B are parameters which are usually determined experimentally [36,37,55,57,88,89]. Of course, this is a simplification, as the measured plots of γ are usually more complex and can not be solved analytically. Nevertheless, Equation (4) provides us an interesting and testable formula. Pressure dependence of γ is less clear, with some researchers proposing a formula similar to Equation (4):

(5) $$\left(p\right)=Ce^{D/p},$$

where C and D are again experimentally determined parameters [88,90]. The models used by various researchers will be explained in the last section of the next chapter.

**Figure 6:**
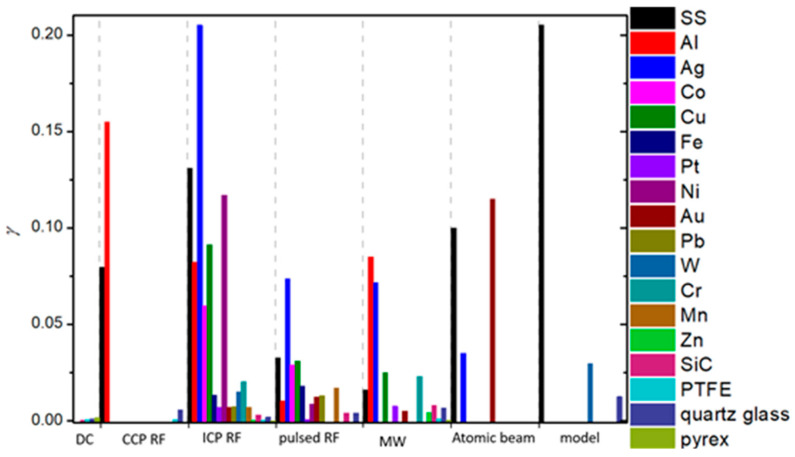
Comparison of average values of *γ* determined using different experimental and theoretical approaches.

To summarize, a reader must be aware that the use of different discharges presented in this chapter and different methods for the determination of recombination coefficients, which will be presented in the next chapter, may have a strong influence on the reported values. In Figure 6, we show a comparison of average values of γ for oxygen recombination on various materials determined in different plasma systems [37,38,39,40,42,43,44,45,46,47,50,51,52,53,54,55,56,57,58,59,60,61,62,63,64,65,66,69,70,71,72,73,74,75,76,77,78,79,80,81,82,83,84,85,86,87,88,89,90,91,92,93,94,95,96,97,98,99,100,101,102,103,104,105,106,107,108,109,110,111,112,113,114,115,116,117,118,119,120,121,122,123,124,125,126,127,128,129,130]. We can observe that, in general, larger values were reported in RF plasmas (especially ICP), which are incidentally also the most commonly used plasma systems. In Table 1, we provide a brief overview of the advantages and disadvantages of various types of plasma discharges.

## 3. Methods for *γ* Determination

Different methods for the determination of recombination coefficients were used by various researchers and will be presented below. These methods can be divided into several categories: calorimetry, emission spectroscopy, actinometry, NO titration, induced fluorescence, Wrede–Harteck gauges, and other experimental methods and modeling. Some of these methods were also used in conjunction with other methods.

The samples used for measuring the surface recombination coefficients were either placed into the oxygen (or oxygen-containing) plasma or in the afterglow. In the case a sample is facing plasma, its surface will charge negatively against the plasma because the mobility of plasma electrons is much larger than the mobility of ions. The negative surface potential will form a sheath between the negatively biased sample and the plasma. The sheath voltage will assure for equal flows of negatively and positively charged particles and, thus, stable conditions. Obviously, the voltage depends on the electron temperature in gaseous plasma. The positively charged ions entering the sheath are accelerated toward the sample and bombard the surface. The ions thus supply kinetic energy, which is shared with the surface atoms upon impinging. The kinetic energy in most plasmas reviewed above will be roughly 10 eV in the case of collision-less sheaths and below this value in cases where the sheath thickness is not much smaller than the mean free path. The energy supplied to surface atoms by surface bombardment with positively charged ions may influence the recombination coefficient.

### 3.1. Calorimetry

**Figure 7:**
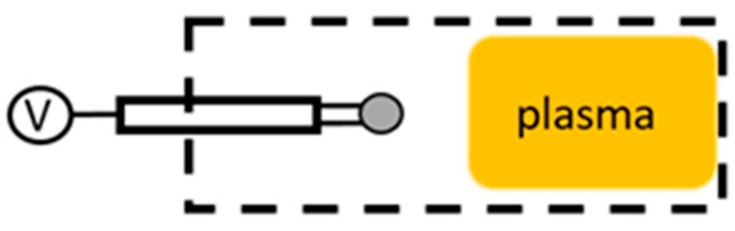
Schematic of a typical calorimetric probe for measuring the O-atom density via the heat dissipated on the surface of the probe, which enables the determination of the recombination coefficient.

One of the most often used methods is calorimetry which is based on measurements of heat exchange. These measurements can be performed by catalytic probes (thermocouple, fiber optic), other methods utilizing thermal resistivity (thin film resistance thermometers, thin film heat-transfer gauges), pyrometry, and other calorimetric detectors. In all cases, an O-atom-sensitive material is introduced into the plasma reactor, and the change in temperature due to the surface recombination of O-atoms is measured. In Figure 7, we show an example of using a catalytic probe for the determination of the recombination coefficient because of heat dissipation on the surface of the probe during the recombination of O atoms.

In a study by Mozetič and Zalar [59], a thermocouple probe was used to measure neutral atom density and later replaced by a thermocouple probe attached to the investigated material, which was stainless steel. Comparison of measured data between the two probes helped determine the recombination coefficient of stainless steel as 0.07 in the regime of 10–100 Pa and 400–700 K. In a similar study by Mozetič and Cvelbar [92], recombination coefficients of several metals were determined using a thermocouple probe. In addition, discharge was monitored with a Langmuir probe for electron energy and density detection and an optical spectrometer. The latter was used in NO titration to confirm the readings of the catalytic probes. Another study with a similar experimental procedure was performed for a niobium surface by Mozetič et al. [91], with the result γ=0.09, constant for pressures between 100 and 400 Pa and temperatures between 420 and 620 K. Yet, another study by Mozetič et al. [49] brought results for carbon nanowalls, a carbon-based nanomaterial best described as shredded ribbons of graphene growing perpendicular to a surface. This exotic material exhibited an especially high recombination coefficient with γ=0.59 at 50 Pa and 300 K.

In a paper by Zaplotnik et al. [76], recombination coefficients for polyethylene terephthalate (PET), polystyrene (PS), and polytetrafluoroethylene (PTFE) were determined with the help of two catalytic probes. One probe had a catalytic tip, while the other had a tip made of the studied polymer, and the responses of both probes were compared in order to determine the recombination coefficient. A similar twin catalytic probe approach was used by Cvelbar et al. [93] to determine the recombination coefficient of various metals, with a minor difference in the catalytic probes, because the heat dissipated on a surface due to recombination of O-atoms was monitored through an optical fiber instead of a thermocouple. The measured densities of neutral atoms were confirmed with NO titration, while the energy and density of electrons were measured with a Langmuir probe.

A method used often in conjunction with calorimetry is the side-arm method [57,94,95,96,103,104]. In such a method, atoms are created in the discharge chamber and then let through a small orifice to a tube-shaped side-arm chamber. The gas diffuses along the length of the tube, with atom losses occurring at the walls of the side-arm. In a study by Drenik et al. [94], such a side-arm method was used, with the walls of the side-arm covered in amorphous carbon. The decrease in neutral atom density along the arm was measured with a fiber optic catalytic probe, and both hydrogen and oxygen recombination was determined. Another work by Drenik et al. [95] used the same approach for the oxygen atoms on aluminum surfaces, both clean and with a layer of deposited carbon. In Drenik’s thesis [96], this same approach was used to determine γ for graphite and different deposits of amorphous carbon. The measured γ of amorphous carbon deposits was not very high (at around 0.001), but was more than 10 times higher for polished graphite at 0.05. However, this is still nowhere near the reported γ=0.59 of nanostructured carbon reported by Mozetič et al. [49]. Comparing γ of different forms of carbon hints at the impact surface morphology can have on surface recombination.

Linnett and Marsden [58] also used a catalytic probe to measure the drop in neutral atom density inside a side-arm with deposits of various salts and oxides. They found that the γ of the studied materials increased drastically with an increase in surface temperature, with some materials exhibiting a rise in several orders of magnitude. For example, the γ of KCl rose from 0.00008 to 0.01 in the temperature range from 300 to 700 K. Very similar behavior was noticed for LiCl, but not for oxides such as PbO, MoO_3_, quartz, and Pyrex, where an increase in γ in respect to surface temperature was not as drastic.

The same method, albeit modified, was used by Greaves and Linnett [56] when measuring γ for several groups of materials: metals, non-metals, oxides, and halides. It was found that metals exhibited the highest γ, with Ag and Cu showing the highest γ at $\gamma_{Ag}=0.24$ and $\gamma_{Cu}=0.17$. This study also confirmed the formation of an oxide layer on metals during exposure to oxygen, which eventually stabilizes the recombination process. In a paper by Goulard [87], the results of Linnett and Marsden [58] were compared to a theoretical model for surface recombination and to results obtained in a shock tube, where the neutral O-atom density was measured with a thin copper film heat-transfer gauge. The $\gamma_{Cu}=0.4$ reported is somewhat high, which might be due to a different type of discharge used.

Elias et al. [97] determined the recombination coefficient of Pyrex ($\gamma_{pyrex}=0.000077$) with a movable detector, which consisted of a platinum wire coated in a catalytic material. An electrical current was applied through the platinum wire, which kept the detector at a constant temperature, measured by its resistance. Upon recombination of atoms on the surface of the detector, the current was lowered so that the resistance (and temperature) of the detector remained the same. The energy dissipated due to surface recombination was calculated from the drop of the electrical current needed to sustain a constant temperature.

Another use of catalytic probes in the side-arm was reported by Dickens and Sutcliffe [57], who measured γ for various metal oxides and quartz. In their findings, they noticed discontinuity in the temperature dependence of γ for several materials, as well as a general trend, where γ was highest for p-type oxides, lower for n-type oxides, and lowest for insulating oxides. They also found that for certain oxide films, conductivity rose with exposure to atomic oxygen. However, not all oxides exhibited such behavior.

Hartunian et al. [98] also used a catalytic probe having a fast response time (less than 1 μs). This allowed instantaneous measurements of changes in surface temperature. The probe had a thin platinum resistance thermometer in the shape of a sphere or cylinder at the tip, which was coated in a layer of silicon oxide, a dielectric. This dielectric layer was, in turn, coated with a catalytic film. γ for quartz, Pyrex, silver oxide, nickel oxide, aluminum oxide, and spinel (MgAl_2_O_3_) was measured in this setup at room temperature, and pressures ranging from 7 to 15 Pa were noted. Spinel and silver oxides were proved to be the most efficient catalytic materials.

A very similar approach was used by Myerson for measuring the γ of gold, platinum, palladium, and TiO_2_ [64] and for measuring the γ of copper, iron, nickel, aluminium, gold, and silver [63]. Measurements were carried out in the pressure range from 400 to 1300 Pa, with the surface temperature of materials kept around room temperature. While most noble metals were not found to be good catalysts for oxygen recombination, silver was again the material with the highest γ.

Another use of calorimetry was presented by May and Linnett [99] in conjunction with Wrede–Harteck gauges and the effusion method, where gaseous plasma is effused through a small orifice into another chamber, with a detector placed immediately behind said orifice. Through surface recombination, atoms heat up the detector, and the change in temperature is detected via thermal resistivity. Again, silver proved to be the most catalytic material, followed by copper and then by chromium and gold. However, those measurements were carried out in a limited range of surface temperatures (300–400 K) and at pressures of around 2 Pa.

Measurements of reaction-cured glass (RCG), an exotic material used as a coating for spacecraft reentering the planetary atmosphere, were performed by Scott [100]. Samples of RCG were attached to water-cooled holders equipped with platinum thermocouples. A referential nickel thermocouple probe was installed to measure the neutral atom density in the vicinity. It was shown that with higher surface temperatures (around 1500 K), the γ of RCG increased by a factor of 3 compared to room temperature (from 0.008 to 0.023).

Another use of a moving platinum thermocouple probe to measure the neutral atom density of gas diffusing along a tube was employed by Kim and Boudart [101] when determining γ for oxygen, hydrogen, and nitrogen atoms on quartz glass. The quartz walls were kept at the desired temperature by being submerged in a constant temperature bath. Therefore, a wide range of temperatures of quartz was achievable, from 200 to 1200 K. The γ of quartz glass increased at higher temperatures for all three gases.

Gordiets et al. [102] used thermocouple probes in combination with electrostatic probes and NO actinometry to measure the γ of Pyrex for oxygen, nitrogen, and their mixtures. While NO actinometry was used to measure neutral atom density and electrostatic probes to measure electron density, the γ of Pyrex was determined by the heat transferred from plasma to the walls. An interesting observation was reported: γ for both oxygen and nitrogen changed when the two gases were mixed and increased with an increasing percentage of oxygen in the gas mixture.

The popularity of using a catalytic probe in a side-arm as a method of determining γ is again demonstrated by Stewart [103,104]. In his work, he studied thermal protection materials (Pyrex, SiC, and other, more exotic silica-based materials, as well as carbon-based coatings) as candidates for spacecraft shielding during planetary reentry. Stewart also employed a heavy-duty electric furnace to heat the materials to very high temperatures, essentially reaching the upper limit of the materials (in some cases, approximately 2000 K). Interestingly, most of those materials exhibited an increase in γ with temperature up to a certain point. At higher temperatures, γ seemed to decrease with increasing temperatures. Additionally, noteworthy is the use of laser-induced fluorescence to corroborate the values of neutral oxygen atom density.

Bykova et al. [105] used a continuous-flow stationary calorimeter while testing the γ of different heat-shield candidate materials at high temperatures. Coated molybdenum, quartz glass, and Pyrex were attached to thermocouple probes, and the temperature response of the probes was measured in oxygen plasma. With the help of a mathematical model, γ was determined. Quartz glass catalyticity was increased with temperature, while Pyrex exhibited a decline in catalyticity with surface temperature. Molybdenum was not studied beyond room temperature, where $\gamma_{Mo}=0.0048$.

Another comprehensive study of γ for various metals (copper, silver, cobalt, zinc, nickel, gold, and steel) was reported by Cauquot et al. [106]. In this study, samples of metals were placed in stainless-steel heater cartridges and kept at room temperature and a pressure of 300 Pa. The temperature was measured with a platinum resistance temperature detector probe, and the neutral atom density was measured by NO titration. Copper and silver were again determined to be the materials with the highest γ for the recombination of oxygen atoms.

In an experiment by Šorli and Ročak [107], γ was determined for nickel with the help of two catalytic probes, the first having a disk-shaped nickel tip, and the second having a tube-shaped nickel catalyst. The tube was placed next to a narrow glass tube so that all (or at least the vast majority of) the atoms passing through it recombined. Afterward, the tube was removed, and the neutral atom density was measured with the second probe. Comparing the response of both probes yielded a constant $\gamma_{Ni}=0.27$ of nickel for a range of temperatures from 500 to 1100 K and the pressure ranging from 10 to 100 Pa. Along with that, the γ for Pyrex was determined as $\gamma_{pyrex}=0.00019$ at room temperatures and in the same pressure range as before.

Zheludkevich et al. [108] studied the oxidation of silver and silver oxide and determined for both that $\gamma_{Ag}=\gamma_{Ag_{2}O}=0.1$ at extremely low pressures (about $10^{-7}\mathrm{Pa}$) and in the temperature range 373–673 K. This was determined by measuring the time dependence of electrical resistance of silver filaments. Experiments were reproduced three times without major discrepancies. Researchers noted that at temperatures higher than 673 K, the oxide layer became unstable, yielding unreliable results.

Kolodziej and Stewart employed both thin film calorimeters and thermal capacitance (slug) calorimeters to measure heat fluxes in their system. The walls of the experimental system were made of Pyrex, and their temperature was also measured by IR pyrometry. All the acquired data were used in conjunction with a mathematical model to determine γ. They found that for Pyrex, the increase in γ for oxygen can be of a whole order of magnitude and five times less for nitrogen when working at temperatures above 1000 K and pressures between 800 and 3000 Pa.

Another use of both theoretical and experimental results was presented by Kolesnikov [78]. He used water-cooled stationary heat flux probes to study the experimental parameters of oxygen and nitrogen recombinations on quartz glass. He later used a tethered particle motion (TPM) model to determine $\gamma_{quartz}=0.003$ at $10^{4}\;\mathrm{Pa}$ and 300 K. The TPM model was used to calculate the movements of physisorbed atoms on the surface of the quartz glass, as surface mobility of adatoms seems to play a key role in the heterogenous surface recombination of neutral atoms.

Herdrich et al. [109] determined the γ of oxygen atoms for a ceramic material known as PM1000. The material is made of carbon, nickel, chromium, iron, aluminium, titanium, and yttrium oxide and is considered a thermal protection material for spacecraft during planetary reentry. The material was put inside pure oxygen plasma with a heat flux sensor at the stagnation point (the point of the material which takes the brunt of the flowing gas). This sensor produces an electric signal proportional to the applied heat flux to the sensor’s surface. The temperature of the material was also monitored with an optical pyrometer, while the flow of gas was measured with a Pitot probe. At 800 Pa, the γ of PM1000 increased from 0.17 to 0.21 in the temperature range from 1500 to 1610 K.

A later study by Steinbeck et al. [110], using the same facilities and techniques as Herdrich et al. [109], determined γ for pre-oxidized tungsten, PM1000, and its preoxidized form, SiC, and preoxidized SiC. At 83 Pa and 1371 K, the γ for pre-oxidized tungsten was 0.035. PM1000 in the temperature range 1499–1611 K at similar pressures exhibited a slight increase in γ from 0.206 to 0.233. Under the same conditions, an even higher γ=0.313 was observed for pre-oxidized PM1000. Quartz glass exhibited a two-times increase in γ when temperature increased from 1180 to 1650. With SiC, γ seemed to drop with increasing temperature (1300–2000 K) from 0.085 to 0.009, but remained more or less the same in that temperature range for pre-oxidized SiC, albeit with a slightly higher value of $\gamma_{oxid.\;SiC}=0.114$.

Park [86] used calorimetry in conjunction with a mathematical model to determine the γ of neutral oxygen atoms on copper and cupric oxide in a gas mixture of oxygen and argon. In his experiments, a thin film heat-transfer gauge was used to measure the heat transferred from plasma to the material through heterogenous surface recombination. The experiment was performed at a higher pressure (14,000 Pa) and room temperature, with the results providing $\gamma_{Cu}=0.4$ and $\gamma_{CuO}=0.0029$. The use of the same facilities to determine γ for copper at atomic oxygen partial pressures from 13,410 Pa to 25,620 Pa and at room temperature was described by Yang et al. [40]. γ decreased with rising pressure from 0.0022 to 0.0213.

### 3.2. Emission Spectroscopy

**Figure 8:**
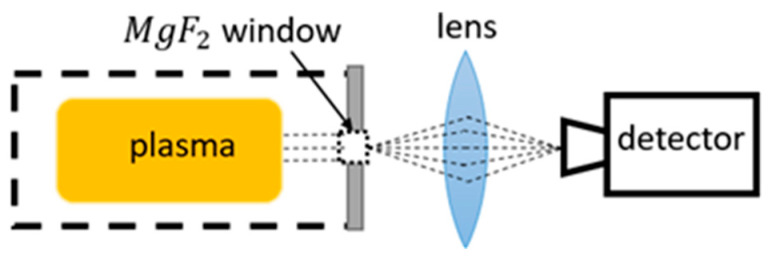
Schematic of an emission spectroscopy technique.

In methods utilizing emission spectroscopy, the relaxation of excited plasma particles is monitored. All particles tend towards their lowest possible energy, which is at their ground state. Excited particles, which are created in the plasma discharge, sooner or later de-excite to their ground states, releasing energy. Some of that energy is released through photons, which are usually in the optical or VUV range [96]. The schematic of this technique is shown in Figure 8.

Mange et al. [111] utilized the VUV emission spectroscopy in measuring the γ of Pyrex in the pressure range of 3–667 Pa. The decay of oxygen atoms along a Pyrex tube at room temperature was measured through a $MgF_{2}$ window positioned in the afterglow, resulting in $\gamma_{pyrex}=0.0024$. This was possible due to the high intensity of the three resonant atomic triplet lines which were analyzed in the range of wavelengths from 120 to 210 nm.

In a series of articles by Cartry et al., time-resolved VUV absorption spectroscopy was used to determine γ of quartz in oxygen discharges [44], and the experimental data was later used in a mathematical model [45]. Three atomic oxygen lines at 130.217 nm, 130.487, and 130.604 were monitored. At 133 Pa and room temperature, $\gamma_{quartz}$ was experimentaly determined to be around 0.0001–0.0004, with a very similar value of 0.0005 provided by the mathematical model.

### 3.3. Actinometry

Actinometry utilizes optical emission spectroscopy to measure neutral atom densities. A known and low quantity of an actinometer, which is usually a noble gas (argon is used most frequently), is introduced into the experimental system where oxygen plasma is sustained. In the optical spectra, the intensity of the actinometric lines is compared to the intensity of the studied gas lines. Since the quantity of the actinometer is known, the ratio of intensities can be linked to the neutral atom density of the studied gas. A prerequisite for this method to work is that the emitting state of the actinometer needs to be at around the same energy as the emitting state of the gas in question.

In a study by Cartry et al. [77], the γ for oxygen atom recombination on a quartz glass surface was determined. Interestingly, two two sets of values were presented, one set for the fast-decaying atoms inside the discharge and one set for the slow-decaying atoms in the afterglow. Both sets were operated at pressures of 67, 133, and 267 Pa. The coefficients were measured for the quartz glass surface at room temperature. Measurements in the discharge provided higher values of $\gamma_{quartz}=0.04-0.028$, which decreased with increasing pressure. On the other hand, afterglow measurements provided $\gamma_{quartz}=0.00019-0.0005$, which increased with increasing pressure. The discrepancy was explained by the creation of additional active recombination sites on quartz through ion bombardment in the discharge area. This is one of a few scientific articles which addresses the role of charged particles (and perhaps also VUV radiation) on the surface recombination of oxygen atoms.

Krištof et al. [112] used actinometry for the determination of γ for quartz (0.0039), PET (0.00093), PTFE (0.00066), and mica (0.0012) at room temperature and pressures 150–350 Pa. Argon was used as the actinometer and emissions were observed along a 10 cm long afterglow chamber, with light being focused by a lens into an optic fiber connected to a spectrometer.

Booth and Sadeghi [69] used actinometry to study plasmas of pure oxygen and mixtures of oxygen and fluorine. Although they did not determine γ, they did determine the sticking coefficient (α) of oxygen atoms on the reactor wall, made of stainless steel. Their results show that in pure oxygen plasma, $\alpha_{O,\;SS}=0.5$, but is significantly lowered to $\alpha_{O+F,SS}=0.09$ in mixtures of oxygen and fluorine. Their experiments were performed at 3 Pa, and the reactor walls were at room temperature. The emission of oxygen atoms was observed at 844 nm and compared to the emission of argon atoms at 750 nm.

Pagnon et al. [113] measured the γ for quartz at room temperature, and observed an increase from 0.00002 to 0.0024 with increasing pressure from 50 to 300 Pa. Two spectral lines of oxygen, one at 844 nm and the other at 777 nm, were monitored along with the 750 nm argon line. The results of the actinometric method were also compared to those achieved using VUV spectroscopy, with a high degree of agreement between the two methods.

In a series of papers by Balat-Pichelin et al. [79,80,81,82,83,84], actinometry was utilized in conjunction with other methods to determine the γ of various materials. The 844 nm oxygen line and the 824 nm argon line were monitored in the discharge area. The studied materials included different steel alloys, aluminum oxide and its alloys, quartz, β-cristobalite quartz, SiC, a mixture of quartz and SiC chromium oxide, and various exotic alloys of zirconium (namely Y_2_O_3_ stabilized zirconia (YSZ), CaO stabilized zirconia (CSZ), MgO stabilized zirconia (MSZ), and ZrB_2_-based materials). All of the listed materials were tested at pressures ranging from 100 to 1000 Pa and at relatively high surface temperatures (from 1000 to 2000 K), and all of the materials exhibited a rise in γ with increasing surface temperature, allthough in various amounts. All materials were studied as potential candidates for thermal shielding of spacecraft during planetary reentry. Therefore, to reach higher temperatures, the materials were heated using a solar furnace, and their temperature was monitored with an IR pyrometer. A calorimetric probe was used in the absence of materials to measure neutral atom densities at the same spot under the same experimental conditions. The results of the studies showed that aluminum-based alloys were the best catalysts, followed by steel alloys as well as zirconium alloys. Chromium oxide behaved similarly to quartz glass and SiC-based materials, remaining inert, with γ growing considerably at the high end of the temperature range (around 2000 K).

Guyon et al. [65,66] used actinometry along with NO titration to measure γ for various semiconductors at 110 Pa from their room temperature to the upper limit, where the materials would remain stable. A heater cartridge was used to heat the materials. The emission was observed through a collimator connected to a spectrophotometer through an optical fiber. A linear correlation between activation energy needed for heterogenous surface recombination and the energy gap of p-type oxide semiconductors was discovered upon studying MnO, CoO, PbO, and Sb_2_O_3_. An increase in γ was also noticed with the decreasing energy gap of p-type oxide semiconductors. However, no correlation was found between the gap energy of n-type semiconductors (WO_3_, BaTiO_3_, TiO_2_, CaTiO_3_, Al_2_O_3_, SiO_2_, Fe_3_O_4_, and SiC + SiO_2_) and γ. A logarithmic correlation between the activation energy of heterogenous neutral oxygen atom recombination and the density of active sites on n-type semiconductors was reported. All of the studied semiconductors exhibited an increase in γ with increased surface temperature [65,66].

A time-resolved actinometric method for the determination of heterogeneous loss of O, H, F, and CF_2_ radicals on a quartz surface was proposed by Lopaev and Smirnov [43] and expanded upon by Lopaev et al. [42]. The loss of oxygen atoms was measured from the radial variation of the dissociation degree of oxygen. At higher pressures, the actinometric method had to be corrected, which required the spatial distribution of the reduced electric field to be known. In their findings, room-temperature quartz glass was examined first in the pressure range of 13 to 400 Pa [43] and later from 600 to 6600 Pa [42]. The results for the first pressure range provided a constant $\gamma_{quartz}=0.00205$, while $\gamma_{quartz}$ grew to 0.003 at higher pressures.

Macko et al. [47] also employed the time-resolved actinometric method to determine the γ of Pyrex in respect to the surface temperature in a pulsed discharge system. The decay of oxygen atom density after each pulse of the discharge was studied to determine the γ of the Pyrex walls of the reactor. Argon was used as the actinometer, and the line at 750 nm was monitored along with two oxygen lines at 777 and 844 nm. In the temperature range from 77 to 460 K, γ increased by two orders of magnitude, from 0.0004 to 0.016. In order to achieve lower temperatures, the tube was cooled with liquid nitrogen. Higher temperatures were obtained using a resistance heater. No pressure dependence of γ was observed in the range from 66 to 626 Pa.

Another use of the time-resolved actinometric method was documented by Bousquet et al. [114] when determining the γ of stainless steel plasma reactor walls. Along with that, a Langmuir probe was used to measure electron density and electron energy distribution. A gas mixture of oxygen and hexamethyldisiloxane (HMDSO) was used, along with pure oxygen, CO_2_, and H_2_O. The γ of oxygen atoms was found to be around five times higher in pure oxygen plasma (0.09) than in pure CO_2_ plasma (0.02), which was attributed to the competition of CO and O radicals for the same surface adsorption sites. In pure H_2_O and mixed oxygen and HMDSO plasmas, the recombination of oxygen atoms was further diminished by the adsorption of OH radicals onto the wall surface. Ion bombardment during the treatment seemed to create more adsorption sites, which increased γ. This observation is similar to that proposed in the paper by Cartry et al. [77].

Rakhimova et al. [54] used actinometry to determine atom loss coefficients of hydrogen and oxygen atoms on nanoporous dielectrics, quartz, and PTFE. Dielectric materials used were SiOCH with porosity between 24 and 33%. Experiments were performed at pressures of 400 Pa and 1300 Pa, and all the materials were kept at room temperature. The hydrogen line at 656 nm and the oxygen line at 777 nm were monitored, along with the argon line at 750 nm. After exposure to plasma, the amount of oxygen and hydrogen atoms absorbed into the sample surface was determined by Fourier-transform infrared (FTIR) spectroscopy. Atomic oxygen was shown to be a prominent remover of CH_3_ groups from the surface of SiOCH, while hydrogen failed to break the $\mathrm{Si}-{\mathrm{CH}}_{3}$ bonds, limiting the damage of hydrogen plasma to the samples. The γ of oxygen atoms was calculated to be 0.0006 for PTFE, 0.0012 for quartz, 0.0038 for the SiOCH with 24% porosity, and 0.0044 for the SiOCH sample with 33% porosity. Therefore, the larger porosity increased the recombination coefficient.

Another study of stainless steel reactor walls and their interaction with oxygen plasma was reported by Tsutsumi et al. [51]. Using energy-resolved actinometry, they determined $\gamma_{SS}=0.01$ at 40 Pa and 300 K. The use of energy-resolved actinometry allowed simultaneous measurements of the radial distribution of oxygen dissociation degree and electron temperature. Two oxygen lines at 777 and 844 nm were monitored along with the 750 nm argon line using phase-resolved optical emission spectroscopy to determine the radially resolved oxygen dissociation degree and the electron temperature.

Morillo-Candas et al. [115] compared the actinometric method with the high-resolution two-photon absorption laser-induced fluorescence for the determination of γ for oxygen atoms on Pyrex. Plasmas were sustained either in O_2_ or CO_2_ discharges. The latter method will be explained in the next section of this chapter. Pressures ranged from 27 to 667 Pa, while the surface temperature of Pyrex was kept between room temperature and 410 K. The increase in gas temperature was shown to increase γ, which was twice as high in pure oxygen plasma (0.00034–0.0011) compared to the γ of CO_2_ plasma (0.00022–0.00065). Good agreement between the two complementary techniques was reported [115].

### 3.4. Laser-Induced Fluorescence

Laser-induced fluorescence (LIF) utilizes selective excitation of ground-state atoms with a laser. Once excited by the laser, the atoms release photons through fluorescence and assume a lower excited state. A laser set to 130 nm is used to excite the oxygen atoms from the ground state ($2p^{4}3P$) to the excited state (3p3P), and the fluorescence deexcitation of oxygen atoms at 845 nm causes the atom to relax to the state $3p^{3}S$ [116]. The density of neutral atoms in the ground state can be determined by measuring the absorption of laser light and fluorescence.

**Figure 9:**
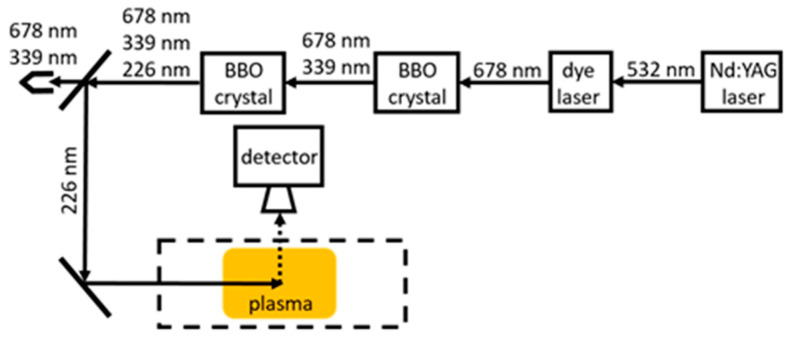
Schematic of a typical TALIF setup for measurements in oxygen plasma. Firstly, an Nd:YAG laser (in this case, with its second harmonic at 532 nm) is used to pump a continuum dye laser. This laser light is frequency doubled and tripled using two beta-barium borate (BBO) crystals. Lastly, the laser light is filtered and focused on the plasma, with the detector for the fluorescent light at a right angle.

Since lasers of such short wavelengths are not feasible [116], some workaround solutions must be incorporated. If two photons of larger wavelengths are absorbed simultaneously, laser-induced fluorescence can be achieved. This method is called the two-photon absorption laser-induced fluorescence (TALIF). In the case of oxygen, two photons with a wavelength of 226 nm are absorbed (Figure 9), while for hydrogen, two photons with a wavelength of 205 are needed to achieve fluorescence at 656 nm (Balmer α). Since the collisional cross-section of two-photon absorption is much smaller than for a single photon, a stronger laser source is required. While offering good spatial and temporal resolutions, TALIF is an expensive and rather impractical experimental method.

In a study determining the spatial distribution and temporal evolution of oxygen atoms, TALIF was utilized by Tserepi and Miller [52]. Absolute densities were determined beforehand with NO titration. The spatial distribution of oxygen atoms between parallel plates of an RF discharge proved to be uniform (or very close to). The decay of oxygen atoms was studied to determine γ of stainless steel reactor walls. The 226 nm wavelength of the laser was achieved with an Nd/YAG laser with a wavelength of 1064 nm, which was used to pump a dye laser. That laser light was frequency tripled and focused using a quartz lens. A mathematical model was used along with the experimental results to determine $\gamma_{SS}$, which, at room temperature, gradually decreased from 0.013 to 0.005 at pressures ranging from 10 to 400 Pa.

Matsushita et al. [62] employed TALIF to determine the sticking coefficient (α) of oxygen atoms to a stainless steel surface at room temperature. The sticking coefficient must not be confused with the recombination coefficient, as sticking is only one part of the recombination process. At lower pressures (2 Pa), α was considerably higher (0.4) than at higher pressures of 10 Pa, where α=0.1. The required 226 nm wavelength was produced by the second harmonic generation of a 451 nm dye laser pumped with a XeCl excimer laser.

Another study of gaseous mixtures of nitrogen and oxygen used in plasma discharges was presented by Dilecce and De Benedictis [117]. In their study, TALIF was utilized to measure neutral atom densities and their loss rates, which, in conjunction with a kinetic model, provided the γ for stainless steel. Again, an Nd/YAG laser was used to pump a dye laser to achieve the proper wavelength of laser radiation. The results at room temperature for stainless steel showed a decrease in $\gamma_{SS}$ from 0.006 to 0.002 with rising pressure from 133 to 267 Pa.

Gomez et al. [50] studied the γ of stainless steel, aluminium, silicon, quartz, and polypropylene (PP) using TALIF. A second harmonic at 532 nm from a pulsed Nd/YAG laser was used to pump a dye laser at 572 nm. Afterward, the dye laser output was frequency tripled to produce a 225.5 nm laser light. With temperatures from 400 to 600 K and pressures ranging from 1 to 100 Pa, the γ was calculated for several materials. For stainless steel, $\gamma_{SS}=0.35-0.02$ decreased with increasing pressure. The same results were reported for aluminum, with $\gamma_{Al}=0.3-0.01$ and almost identical results for quartz. On the other hand, the γ for silicon and PP increased with increasing pressure as $\gamma_{Si}=0.02-0.2$, and $\gamma_{PP}=0.02-0.3$. The rather large value of the recombination coefficient for polypropylene might be due to high temperature. Namely, the melting point of polypropylene is just above 400 K.

In a report by Marschall [118], along with reviewing different measurement methods, the side-arm method was used, with TALIF determining the profile of the decay of atoms along the side-arm. Decay was also simulated, and the results were compared. Experiments were performed at 40 Pa with materials remaining at room temperature. The determined γ was 0.016 for stainless steel (SS 304), 0.046 for a constantan alloy with a 45% amount of nickel, 0.0068 for chrome with a 10% amount of chromium, and 0.0053 for platinum.

### 3.5. NO Titration

**Figure 10:**
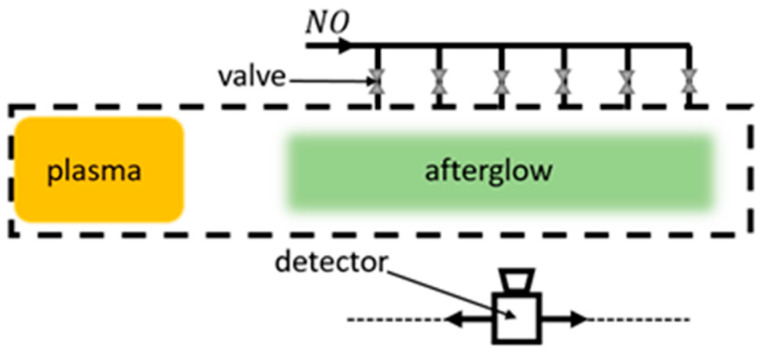
Schematic of a plasma system utilizing measurements with NO titration. NO gas is introduced to the system through a series of valves, and a movable detector monitors the afterglow.

NO titration is the process of adding gaseous NO to the reactor in the afterglow region (Figure 10). In the oxygen afterglow, NO is highly reactive and produces ${\mathrm{NO}}_{2}^{*}$ exciplex molecules. The resulting ${\mathrm{NO}}_{2}^{*}$ molecules are highly reactive with O-atoms, producing NO_2_ molecules and releasing energy via chemiluminescence. The NO_2_ can again dissociate into NO, which can repeat the entire cycle. Due to this process, even a small amount of NO is enough for the rapid depletion of atomic oxygen in the afterglow, and the yellow–green glow due to chemiluminescence can be monitored and linked to the oxygen atom density. A major drawback of NO titration is the poisoning of the reactor with NO and NO_2_ gases. Due to safety reasons, a mixture of argon (around 2%) is used in many practical cases.

In two studies by Kaufman [71,72], the γ for quartz and Pyrex surfaces at room temperature was determined using NO titration. The light intensities in the titration region were measured with movable photomultipliers, which provided the neutral atom density profile along the titration region. For quartz at 67 Pa, γ was determined to be 0.00002, a value much lower than determined in contemporary literature. As for Pyrex, the same value of γ was determined, again low compared to other sources.

In a report by Rosner et al. [119], a study of candidate materials for space shuttle thermal protection systems was carried out. Of particular interest was nickel oxide, with $\gamma_{NiO}=0.1$ at room temperature. Regrettably, the pressure was not reported. One can speculate the pressure was similar to pressures in experimental setups of other authors utilizing NO titration. With that in mind, the pressure in the plasma system could have been between 50–1000 Pa.

Brake et al. [74,75] used NO titration along with a theoretical model when determining the γ of quartz at room temperature. NO gas was introduced into the system through eight pinholes forming a ring in a single cross-sectional plane of the titration chamber, meeting oxygen in a crossflow pattern. A one-dimensional, temperature-dependent mathematical model was in agreement with the experimentally determined oxygen atom density. However, the resulting $\gamma_{quartz}$ provided two different values separated by a whole order of magnitude. From the experimental data, $\gamma_{quartz}=0.0005$, while from theoretical calculations, $\gamma_{quartz}=0.00005$.

A great example of NO poisoning of the reactor due to NO titration was demonstrated by Wickramanayaka et al. [60], with observable changes in γ due to NO poisoning of the surfaces of different materials. At the pressure of 133 Pa and room temperature, the γ of several materials in their original and poisoned state was determined. The γ of poisoned materials was generally lower than before NO poisoning. Results for γ were provided for stainless steel (clean at 0.099 and poisoned at 0.0064), aluminum (clean at 0.0044 and poisoned at 0.0029), aluminum with a layer of Ni + Cr_2_O_3_ (clean at 0.0036 and poisoned at 0.0035), aluminum with a PTFE layer (clean and poisoned at 0.002), copper (clean at 0.026 and poisoned at 0.019), platinum (clean at 0.0027 and poisoned at 0.0016), gold (clean at 0.0032 and poisoned at 0.0019), magnesium (clean at 0.0023 and poisoned at 0.0012), Pyrex (clean at 0.000092 and poisoned at 0.000056), and PTFE (clean at 0.000073 and poisoned at 0.000064). Based on these results, one must take the data obtained by NO titration with some precaution. Namely, titration experiments are often time-consuming, so ample time must be available to poison the reactor walls in the afterglow region.

### 3.6. Wrede–Harteck Gauge

**Figure 11:**
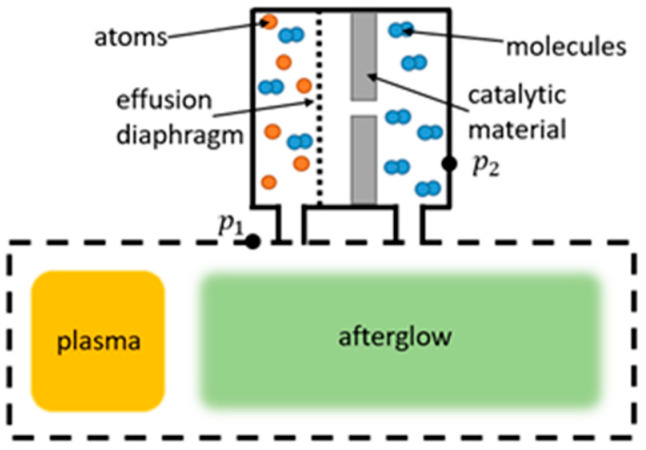
Schematic of a Wrede–Harteck gauge, where $p_{1}$ and $p_{2}$ are pressures measured inside the plasma chamber and inside the Wrede-Harteck gauge, respectively.

Another method used to determine neutral atom densities, and, consequently, γ, is using the Wrede–Harteck gauge. The Wrede–Harteck gauge uses a manometer to measure pressure in a separate chamber connected to the plasma reactor through a small orifice, which allows atoms to pass through. As a result of dissociation, the pressure inside the gauge where atoms are present is larger than in the segment of the gauge where the atoms recombine into molecules because of the presence of the catalytic materials. When in equilibrium, the amount of atoms entering the gauge is balanced by the amount of molecules leaving the gauge. The difference in pressure between the gauge and the system can help us determine the neutral atom density inside the reactor, as shown in Figure 11.

The use of Wrede–Harteck gauges to determine γ was described in the works by Greaves and Linnett [70,120], where the gauge was lined with different materials, and pressure was measured at both ends of the lined gauge using Pirani gauges. At a moderately low pressure of 650 Pa, the γ of several oxides was determined at room temperature. CuO exhibited the highest value at 0.043, while B_2_O_3_ and Sb had the lowest values of 0.000063 and 0.000081, respectively [120]. Quartz glass was examined more closely, with $\gamma_{SiO_{2}}=0.00016-0.014$ growing with rising temperatures from 300 to 900 K and at 16 Pa [70].

Another seminal work for the determination of γ, by Melin and Madix [55], also utilized Wrede–Harteck gauges in conjunction with isothermal calorimetric filaments. Weak temperature dependence of γ was observed for silver and copper, with silver once again proving to be the best catalytic material for oxygen. Cobalt came in next with $\gamma_{C_{O}}=0.075$ at room temperature, followed by copper and its oxide and nickel. Experiments were performed at relatively low pressures ranging from 1 to 4 Pa.

Sabadil and Pfau [121] measured the oxygen dissociation degree in a quartz glass tube at a pressure of 66 Pa using the Wrede–Harteck gauge along with the ozone method, with both methods in good agreement. The ozone method utilizes the association of atomic oxygen with molecular oxygen on a surface cooled with liquid nitrogen into an ozone molecule. The difference in pressure during ozone creation can be linked with the neutral oxygen atom density and, conversely, with γ. For quartz, γ was determined to be around 0.00048.

### 3.7. Mass Spectrometry

This method measures the mass-to-charge ratio of ions. As the gas from the reactor is pumped into a mass spectrometer, the neutral particles are ionized, and the resulting ions are analyzed via separation by mass-to-charge ratio. Examples of mass spectrometers include time-of-flight, magnetic sector, and quadrupole.

In a study by Sjolander [85], the probabilities for reflection, recombination, general surface reaction, and occlusion were calculated for the following materials: gold, nichrome V, aluminum, titanium, silver, and platinum. Measurements were performed at 40 Pa and at room temperature (due to the low density of O-atoms), and the probabilities were calculated using a mathematical model with experimental data as input parameters. The materials were exposed to the oxygen plasma for up to 10 h, which altered their γ. Stainless steel and titanium exhibited an identical rise in γ from 0.04 to 0.16 with increased exposure time to the oxygen plasma, as did silver (from 0.01 to 0.06) and gold (from 0.03 to 0.2). While not explicitly stated as the cause, surface reflectivity of O-atoms decreased with exposure time, which might be due to the surface being slowly cleaned of inert impurities by the force of impact of incident O-atoms.

Singh et al. [122] studied the surface recombinations of oxygen and nitrogen atoms on a stainless steel surface in pure oxygen and nitrogen plasmas with the help of a mathematical model. The input parameters were determined experimentally; the radical and molecule densities were measured with a quadrupole mass spectrometer, while the electron energy distribution function was measured with a Langmuir probe. Appearance potential mass spectrometry was used to ionize and detect either radicals or parent molecules selectively. At the surface temperature of 330 K and a pressure of 5 Pa, the γ for oxygen and nitrogen atoms was determined to be 0.17 and 0.07, respectively.

Another use of experimental data acquired with mass spectroscopy as input parameters in a simulation was presented by Kiehlbauch and Graves [123]. Again, a Langmuir probe was used to determine the electron energy distribution function, along with two separate quadrupole mass spectrometers for the composition of ions and neutral particles. Mass spectrometers were calibrated with argon beforehand, ensuring absolute values of neutral atom densities. Additionally, an optical spectrometer was used to measure the rotational temperature of neutral molecules. In the pressure range from 1 to 15 Pa and surface temperature ranging from 300 to 400 K, the γ of stainless steel reactor walls was determined at a constant value of 0.14.

Kurunczi et al. [124] studied the γ of oxygen atoms on an anodized aluminium surface of the reactor walls. The spinning wall method was employed. The spinning substrate was rapidly rotated (at 40,000 rpm) and periodically exposed to oxygen plasma. The substrate chamber was connected to a mass spectrometer, which analyzed the composition of gas in the system immediately after the interaction of the substrate with the gaseous particles. By varying rotation frequencies, the exposure time of the substrate and the time between exposure and detection by the mass spectrometer were controlled. At 1 Pa and room temperature, the γ of anodized aluminum decreased with increasing rotation speeds from 0.6 to 0.4. These values are very large compared to other reports and could be explained by the very rich morphology of anodized aluminum.

Another study of γ for stainless steel and aluminum surfaces was reported by Hsu et al. [125] in pure argon, mixed argon, and oxygen, as well as mixed argon, oxygen, and chlorine gaseous plasmas. Experimental results complemented a theoretical model. An ion flux probe was utilized to measure the flux of ions to the walls of the reactor, while a Langmuir probe measured electron density and the electron energy distribution function. Separate mass spectrometers were used to measure the composition of ions and neutral plasma particles. In the temperature range from 300 to 400 K and pressures from 1 to 11 Pa, the γ of stainless steel and aluminum were determined to be 0.3 and 0.0001, respectively.

Guha et al. studied the γ of anodized aluminum [126] and performed a separate study of γ for pure and copper-contaminated silicon surfaces [127]. The spinning wall method was utilized along with mass spectrometry and Auger electron spectroscopy (AES) for the detection of desorbing species. The rotation velocity of the spinning wall allowed the control of oxygen atom flux to the surface. At room temperature and pressures of around 1 Pa, the γ of aluminum decreased with increasing oxygen atom flux from 0.06 to 0.04. In the second study, the effect of copper contamination on silicon wafers was studied as well. The reason behind this study is the sputtering of copper to silicon reactor walls during plasma etching of dielectrics. A noticeable decrease in etching rates after some time was linked to an increased oxygen atom sink on the reactor walls due to the catalytic activity of deposited copper. At room temperature and 0.2 Pa, $\gamma_{Si}=0.043$ was noticeably lower than $\gamma_{Si+Cu}=0.069$.

Another use of the spinning wall method was employed by Stafford et al. in combination with a mathematical model to determine γ for oxygen atoms on stainless steel [61] and in a separate study of oxygen and chlorine plasmas and γ for oxygen and chlorine on stainless steel and aluminium reactor walls [128]. In both studies, the use of AES and line-of-sight mass spectrometry helped determine electron density and energy distribution function and the composition of ions and neutral particles. At room temperature and pressure of 0.2 to 3 Pa, the γ of stainless steel was 0.13 for oxygen atoms and decreased with exposure time to 0.09. This was due to the deposition of silicon particles on the material due to the slow etching of the reactor walls. In the second study, $\gamma_{SS}=0.09$ and $\gamma_{Al}=0.05$ were determined under the same temperature and pressure as before. The materials were again coated in a silicon-oxide-based layer, which is the main culprit for lower values of γ for stainless steel.

Donnelly et al. [38] reviewed the methods for studying surface reactions of atoms and molecules. They utilized the spinning wall method to determine $\gamma_{Cu}=0.07$ at 0.02 Pa and room temperature. As with the previously described methods, AES and mass spectrometry were employed for the analysis of electrons, ions, and neutral particles. The experimentally obtained data were used in simulations to calculate γ.

### 3.8. Paramagnetic Resonance

**Figure 12:**
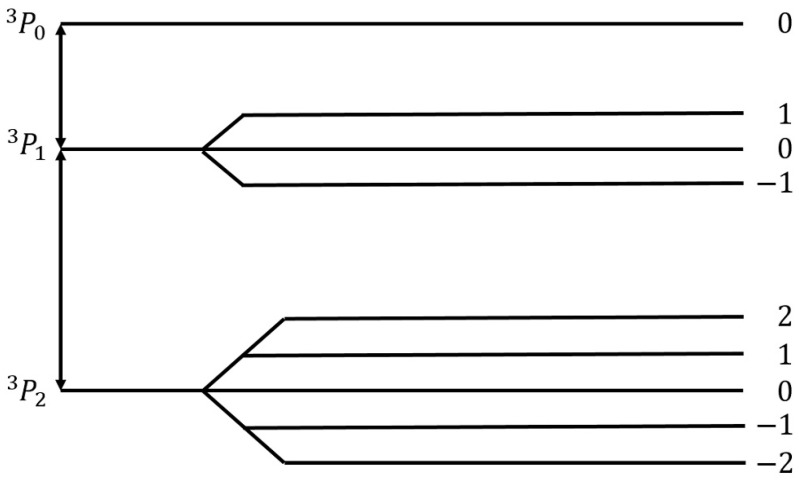
Splitting of the energy levels of an oxygen atom in a magnetic field.

Paramagnetic resonance utilizes the Zeeman effect, the splitting of energies of electronic states in a magnetic field (Figure 12). With an electromagnetic wave with the correct wavelength, electronic transitions can occur between different electronic states. These transitions usually happen in the GHz range of frequencies (wavelengths in the range of centimeters). The relative determination of neutral atom density is possible by monitoring the intensities of the peak in the absorption spectrum. Absolute densities are, however, much harder to determine using this method.

The first study of oxygen recombinations utilizing paramagnetic resonance was demonstrated by Krongelb and Strandberg [129]. They reported a strong paramagnetic-resonance absorption of oxygen atoms. Reactions in the plasma system were monitored by observing the intensity of the absorption. At room temperature and pressures ranging from 100 to 300 Pa, the γ of quartz for neutral oxygen atoms was determined to be 0.00032.

Hacker et al. [73] used paramagnetic resonance along with an isothermal calorimetric detector to determine the γ of quartz glass and platinum. This was conducted by measuring the decay of neutral atom concentration along the flow, which allowed the determination of recombination kinetics. At room temperature and pressures between 150 and 220 Pa, the γ for oxygen atoms on a quartz surface was 0.00004. For platinum, γ increased with temperature from 0.01 to 0.1 in the temperature range of 300–1100 K.

In the paper by Marshall [130], the γ of room temperature Pyrex for oxygen, hydrogen, and nitrogen atoms was studied in the pressure ranges from 133 to 1330 Pa. The γ of Pyrex for oxygen atoms (0.0005) was observed to be almost twice as high as γ for nitrogen atoms (0.0003). Both remained constant throughout the entire pressure range.

Hogan and Burch [131] examined three-body collisions of oxygen atoms and molecules in the gas volume along with the wall recombinations on room-temperature reactor walls coated with a metaphosphoric acid-sodium metaphosphate (NaO_3_P) mixture. The recombinations were monitored with a paramagnetic resonance spectrometer for pressures between 30 and 147 Pa. A liquid-nitrogen trap improved oxygen purity, which reduced the amount of water, CO_2_, and NO_2_ in the system. NaO_3_P was not a very efficient recombinator, with $\gamma_{NaO_{3}P}=0.0000091$ across the entire pressure range.

### 3.9. Theoretical Models

Various authors used different approaches when simulating the recombination of neutral atoms on the surface of materials. Some authors tested these models in experimental setups. We briefly examine some of the works of a few authors and present their results.

In a paper by Hardy and Linnett [37], simulations were based on the semiconductor theory. A connection between γ and conductivity was proposed and the model was applied to three tungsten-oxide-based materials at room temperature. Results show: $\gamma_{Li_{2}WO_{3}}=0.022-0.052$, $\gamma_{Na_{2}WO_{3}}=0.005-0.047$, and $\gamma_{K_{2}WO_{3}}=0.022-0.48$. The method is promising, but not practical for the exact determination of γ.

Seward and Jumper [89,132] used a kinetic model to predict the γ of quartz and reaction cured glass (RCG), which were considered appropriate materials for thermal protection systems of space shuttles during planetary reentry. The results of the model show γ taking a complex form, which was an evolution of Equation (4). The resulting $\gamma_{SiO_{2}}$ showed strong temperature dependence, rising with temperature from 0.0001 at room temperature until a peak of 0.02 at around 1000 K, and then decreasing with increased temperature. This result was reproduced in a separate model, along with similar behavior of RCG with a peak at 1000 K and $\gamma_{RCG}=0.0004-0.01$. The results were in agreement with experimental work reported by other authors.

In the paper by Shibata et al. [53], a relaxation continuum model was used in the numerical analysis of oxygen plasma in a reactor with walls made of stainless steel. The numerical methods were accompanied by experimental results in a CCP discharge at room temperature and 66 Pa. Many values of γ were considered, with two extremes at 0.0001 and 0.5, but the best agreement between the model and the measurements was possible when γ of stainless steel was set at 0.015.

Daiss et al. [133] studied catalytic reactions of oxygen and nitrogen on quartz surfaces. A detailed surface chemistry model for the reactions (adsorption, desorption, and recombination) of plasma particles on the quartz surface was compiled and implemented into Navier–Stokes code. The predicted γ was compared with experimental results of Dickens and Sutcliffe [57], Stewart [103], Scott [100], and Kolodziej et al. [134]. The experimental results were in good agreement with the numerical results, showing an increase in γ with increasing surface temperature up to a peak at around 1000 K and then decreasing rapidly with further increases in temperature.

Quartz was also studied in low-pressure flowing discharges of oxygen and nitrogen by Gordiets and Ferreira [135], who proposed a self-consistent model for bulk and surface kinetic processes. The model was one-dimensional and only applied to DC discharges and afterglows. Kinetics of free electrons, electronic states, chemical and vibrational kinetics, as well as interactions with the wall with respect to gas and wall temperatures were all considered in this model. It was discovered that the γ for oxygen atoms increased with wall temperature and percentage of gaseous oxygen in oxygen–nitrogen mixtures. For nitrogen recombination, γ was shown to decrease with increasing temperatures up to 500 K and then rose with increasing wall temperature.

Cacciatore et al. [136] devised two separate semiclassical models, one for the Eley–Rideal recombination mechanism and the other for the Langmuir–Hinshelwood recombination mechanism. Under the same conditions (wall temperatures from 600 to 1000 K), the theoretical prediction was that the γ of quartz glass would achieve higher values than recombinations that adhered to the rules of the Eley–Rideal mechanism when compared to γ for the Langmuir–Hinshelwood mechanism.

A global volume-averaged model was used to study discharges in a gaseous mixture of oxygen and argon by Gudmundsson and Thorsteinsson [90]. Increasing argon content in the discharge increased the electron temperature, which in turn increased the dissociation of oxygen molecules. The recombination of oxygen on a stainless steel surface was also studied. γ seemed to decrease with increasing pressure (0.1 to 20 Pa) from 0.5 to 0.1. This seemingly supports the theory that γ is negatively impacted by increasing pressure in the system.

Marinov et al. [88] utilized two approaches when modeling surface reactions of gaseous plasmas: the coarse-grained deterministic and the kinetic Monte Carlo methods. It was found that the kinetic Monte Carlo method produced more reliable results but was computationally more demanding. Both the Eley–Rideal and the Langmuir–Hinshelwood mechanisms were incorporated into the models, with γ depending on the total number 1 of active sites on the material surface. In the temperature range from 200 to 400 K, the γ of quartz was determined to decrease from 0.079 to 0.00039.

Experimental results demonstrating a high degree of vibrational excitation of oxygen molecules in oxygen plasmas piqued the interest of Annušova et al. [137], who performed experiments and devised a self-consistent zero-dimensional global kinetic model. The model incorporated electron impacts on ground-state oxygen molecules, the chemistry in the gas phase and the interactions of plasma with the surface. The γ of aluminum was determined at room temperature and pressures from 1 to 10 Pa at a constant value of 0.15, which was considerably higher than most contemporary studies. No surface finish of the aluminum samples was reported.

**Figure 13:**
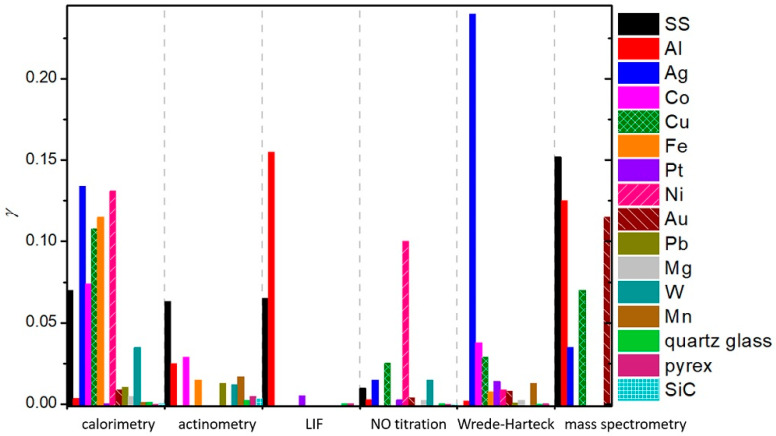
Comparison of average *γ* determined using different measuring techniques.

The results explained throughout the chapter are summarized in Table 2. With all the methods by various authors described, we can now compare the results of their work for various surfaces, which can help us categorize the materials. A comparison of the average γ for oxygen atoms on the material surface, determined using different methods, is shown in Figure 13. Apart from the γ for silver obtained by the Wrede–Harteck method, the largest values were, on average, reported using calorimetry, which is also the most commonly used experimental method. Larger values were also reported in works using LIF and mass spectrometry techniques. Based on Figure 6 and Figure 13, the results thus show that the measured values may depend on the type of discharge system used and on the applied method of measurement. A brief overview of the advantages and disadvantages of the aforementioned measurement methods is presented in Table 3.

## 4. Material Categorization

As explained in previous chapters, different authors used different experimental conditions to determine the recombination coefficients. Some authors reported values for pristine materials, but most for oxidized materials. Many materials will form a thin oxide film upon exposure to oxygen, so it is difficult to ensure an oxygen-free surface. Not surprisingly, the reported results are scattered, sometimes over an order of magnitude or more. The average values are shown in Table 4, which also shows the lowest and largest reported values as well as average values at different temperatures.

**Figure 14:**
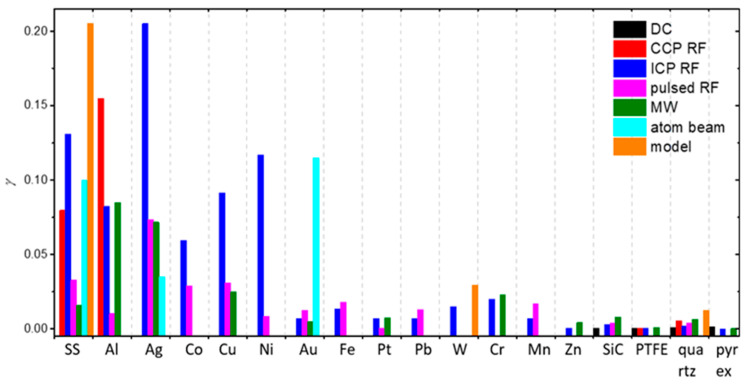
Comparison of average reported recombination coefficients for the most commonly studied materials in various plasma systems.

**Figure 15:**
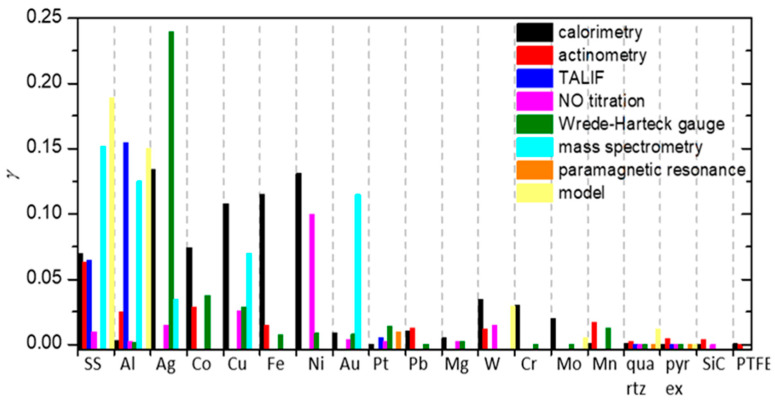
Comparison of average reported recombination coefficients for the most commonly studied materials for different measurement methods.

So much gathered data for γ enables meta-analysis of the reported results. The values reported for a few materials were compared in the hope of finding useful correlations. Based on the determined γ, we can divide the materials into three distinct groups. Materials exhibiting catalytic properties (γ>0.1) were deemed catalytic, followed by semi-catalytic materials (0.1>γ>0.01), and the rest were deemed inert (γ<0.01). Many of the materials were hard to categorize, with their γ evolving with varying parameters, making a material catalytic under certain conditions, only to become inert under different conditions. Therefore, the dividing lines between the categories of materials were mostly blurred and subject to one’s opinion. Nevertheless, this categorization helps tidy up the long list of γ for various materials. A comparison of average reported values of γ for the most commonly studied materials is shown in Figure 14 for various plasma system configurations and in Figure 15 for different methods of measurement. In general, metals have higher γ, with glass materials (quartz glass and Pyrex) having much lower γ. First up, we will examine catalytic materials.

### 4.1. Catalytic Materials

**Figure 16:**
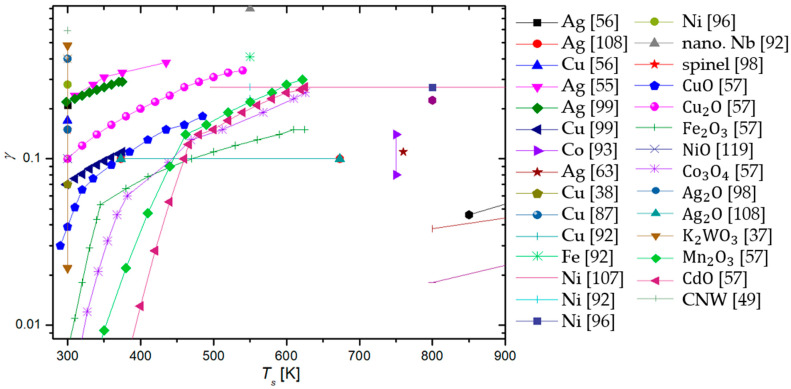
Recombination coefficient (*γ*) of neutral oxygen atoms versus surface temperature ($T_{s}$) for various catalytic surfaces.

**Figure 17:**
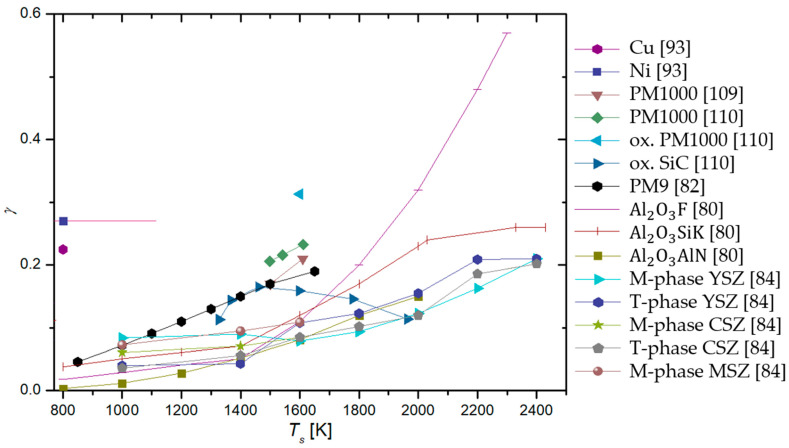
Recombination coefficient (*γ*) of neutral oxygen atoms versus surface temperature ($T_{s}$) for various catalytic surfaces at temperatures higher than 800 K.

The candidate materials most frequently declared as catalytic for oxygen atom recombinations are metals, with Ag, Cu, Ni consistently exhibiting the highest γ in various studies. In Figure 16, γ versus surface temperature is shown for a handful of catalytic materials, while in Figure 17, that same graph is repeated for materials tested at higher temperatures (above 1000 K). In general, γ increases with increasing surface temperature, at least in the range from room temperature to about 2000 K.

**Table 4:** Recombination coefficient values reported for various materials and their composites. Along with the lowest and highest values reported, average values and standard deviations were calculated (where possible). Materials were grouped together with their oxides, as were certain alloys.

Material	Lowest Reported Value	Largest Reported Value	Average Value	Standard Deviation	Average Value (300 K)	Average Value (600 K)	Average Value (1000 K)
stainless steel	0.002 [117]	0.5 [90]	0.103	0.056	0.098	0.123	/
Al	0.0001 [125]	0.6 [124]	0.0948	0.308	0.068	0.101	0.233
Ag	0.015 [106]	0.38 [55]	0.148	0.385	0.114	0.079	0.037
Co	0.00049 [120]	0.25 [57]	0.0763	0.276	0.027	0.157	/
Cu	0.00217 [40]	0.4 [87]	0.0903	0.301	0.0604	0.244	/
Fe	0.0046 [57]	0.41 [92]	0.0685	0.262	0.0203	0.219	/
Pt	0.00055 [64]	0.1 [73]	0.0195	0.140	0.0161	0.0503	0.0503
Ni	0.0008 [57]	0.28 [96]	0.104	0.322	0.0593	0.199	0.270
Au	0.0032 [60]	0.2 [85]	0.029	0.057	0.0347	0.016	0.02
Nb	0.02 [93]	0.09 [91]	0.073	0.030	0.09	0.067	/
Pb	0.00063 [120]	0.09 [58]	0.024	0.030	0.0086	0.09	/
Mg	0.0023 [60]	0.022 [57]	0.00697	0.0835	0.00396	/	/
Cr	0.0002 [57]	0.072 [82]	0.0311	0.176	0.0202	0.0002	0.023
Mn	0.0012 [57]	0.299 [57]	0.071	0.052	0.0104	0.299	/
Zn	0.00034 [57]	0.01 [57]	0.0263	0.162	0.00169	0.01	/
Mo	0.001 [120]	0.02 [58]	0.0082	0.0906	0.0086	0.007	/
Mn	0.0012 [57]	0.299 [57]	0.071	0.267	0.0104	0.162	/
W alloys	0.005 [37]	0.48 [37]	0.0712	0.267	0.0744	0.078	0.035
Zr alloys	0.0073 [84]	0.21 [84]	0.078	0.065	/	0.0415	0.148
Ti alloys	0.0097 [65]	0.078 [65]	0.029	0.022	0.0123	0.0452	/
PM1000	0.0003 [82]	0.313 [110]	0.124	0.101	/	/	0.0718
quartz glass	0.000008 [101]	0.3 [50]	0.016	0.042	0.0111	0.0082	0.0226
quartz crystal	0.0094 [80]	0.1 [80]	0.052	0.043	/	/	0.0497
Pyrex	0.00002 [72]	0.05 [134]	0.0039	0.0097	0.00089	0.00095	0.00057
RCG	0.0004 [132]	0.023 [100]	0.0104	0.0081	0.0004	/	0.009
SiC	0.0003 [104]	0.46 [103]	0.047	0.100	0.00283	0.0268	0.146
PTFE	0.000073 [60]	0.0011 [76]	0.00057	0.00034	0.00057	/	/
carbon	0.0005 [94]	0.59 [49]	0.16	0.25	0.16	/	/

The increasing recombination coefficient revealed in Figure 16 and Figure 17 may be explained by the increasing surface mobility of adsorbed oxygen atoms. As mentioned earlier, one model of surface recombination (Langmuir–Hinshelwood) predicts the association of two adsorbed atoms and the formation of a molecule, which is desorbed from the surface. The surface association of atoms to molecules requires close proximity of two atoms. The average internuclear distance in oxygen molecules in the ground vibrational state is about 0.12 nm. The intermolecular distance is much shorter than the distance between two neighboring atoms in the solid material. Namely, the surface density of atoms in the solid material is roughly 10^19^ m^−2^, so the average distance between two atoms in the solid material is roughly 0.3 nm. It is reasonable to assume at most one adsorbed oxygen atom per atom of solid material, so the distance between neighboring adsorbed oxygen atoms is a few times larger than the distance of oxygen atoms in a stable oxygen molecule. These are average values, and the atoms (both of the solid material and adsorbed O-atoms) oscillate on the surface due to phonon excitations. The oscillation amplitude increases with increasing surface temperature, and thus the probability for association following the Langmuir–Hinshelwood mechanism. Unfortunately, the effect is yet to be addressed by theoreticians who may be able to provide a scientifically spotless model for increasing surface recombination due to increasing oscillations. Equation (4) predicts an exponential increase in the recombination coefficient with increasing surface temperature, but the general behavior, as deduced from Figure 16 and Figure 17, only partially confirms such a behavior.

The discrepancy between the simple theory, as in Equation (4), and the experimental results, as in Figure 1 or Figure 2, may be because of numerous reasons. One trivial explanation is the existence of the Eley–Rideal mechanism, whose temperature behavior is difficult to predict. In general, both mechanisms should be responsible for catalytic surface recombination, but the intensity of surface reactions depends on numerous parameters, such as the density of surface adsorption sites and the surface morphology on the nanometer scale.

It is worth stressing that the surface morphology of many catalytic materials depends on their history, particularly the temperature of materials upon exposure to oxygen plasma. It is known that many metals form nanostructures upon oxidation [139]. The oxidation obviously depends on the surface temperature. The richer morphology on the nanometer or sub-micrometer scale will result in a larger recombination coefficient. The establishment of the rich morphology of surface oxides formed upon treatment of catalytic materials with oxygen plasma is often irreversible. Unfortunately, very few authors reported on the history of the samples before measuring the recombination coefficient for oxygen atoms.

**Figure 18:**
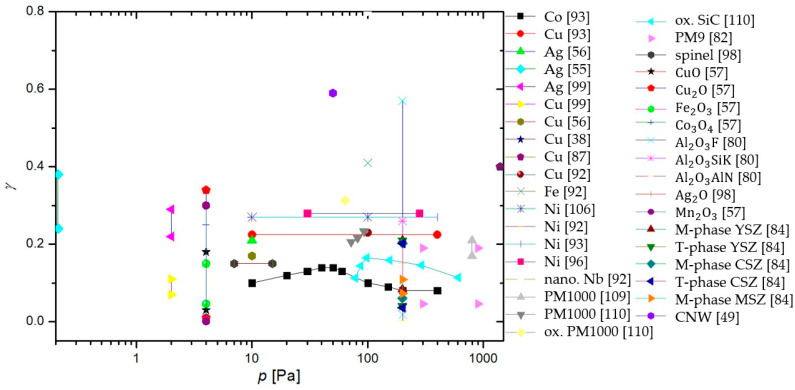
Recombination coefficient (*γ*) of neutral oxygen atoms versus pressure (p) for various catalytic surfaces.

Apart from studying the temperature dependence of γ, we are also interested in the effects of pressure on γ. In Figure 18, the γ of catalytic materials is shown with respect to pressure. However, no discernable trend is noticeable.

### 4.2. Semi-Catalytic Materials

**Figure 19:**
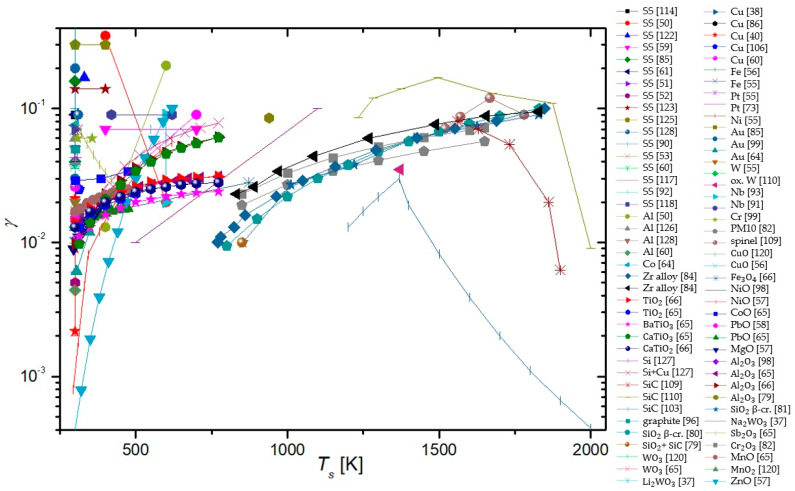
Recombination coefficient (*γ*) of neutral oxygen atoms versus surface temperature ($T_{s}$) for various semi-catalytic surfaces.

Semi-catalytic materials are the most varied category, with materials ranging from various metals and their alloys to different types of glass. This is partly due to semi-catalytic materials being the middle category, with overlap from both catalytic and inert materials. When looking at the surface temperature dependence of γ in Figure 19, it is clear that the relationship is not straightforward. While some materials exhibit a rise in γ with rising surface temperature, others do the opposite, and still, other materials, such as SiC [109,110], C-CAT [104], and spinel [109], behave in more complex ways.

**Figure 20:**
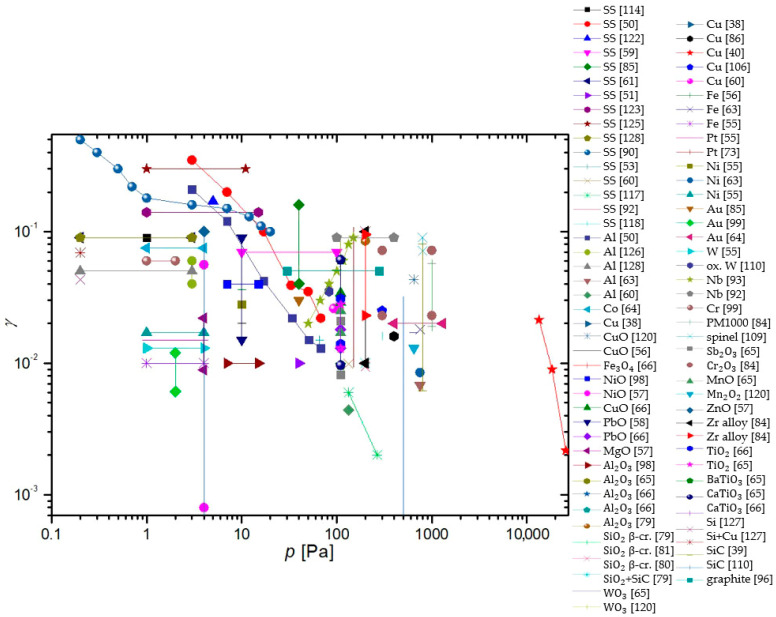
Recombination coefficient (*γ*) of neutral oxygen atoms versus pressure (p) for various semi-catalytic surfaces.

Regarding pressure dependence, the γ of some semi-materials seems to decrease with increasing pressure, but in general, we fail to observe any overarching trends. The results are shown in Figure 20.

### 4.3. Inert Materials

**Figure 21:**
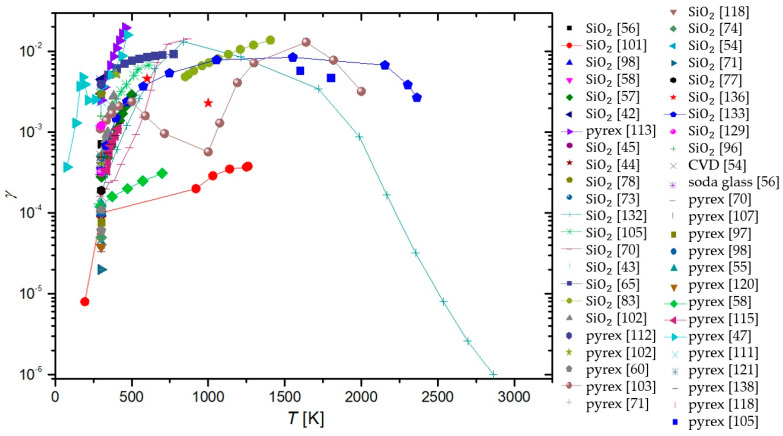
Recombination coefficient (*γ*) of neutral oxygen atoms versus surface temperature ($T_{s}$) for glass surfaces.

**Figure 22:**
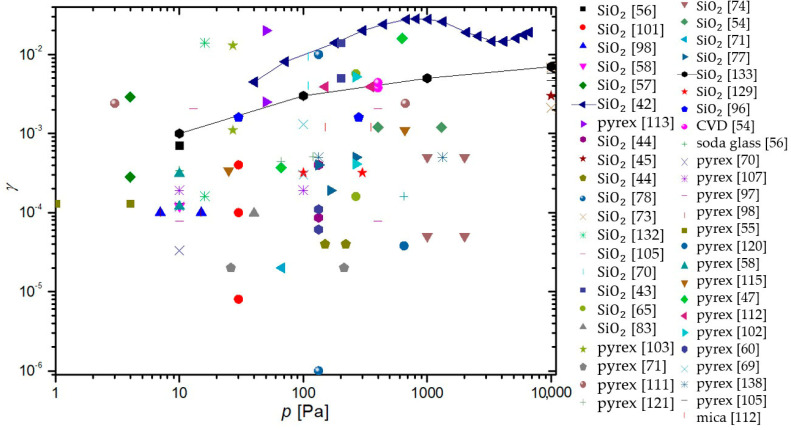
Recombination coefficient (γ) of neutral oxygen atoms versus pressure (p) for glass surfaces.

Materials that have little-to-no interactions with neutral oxygen atoms are categorized as inert. With such γ, only up to 1% of incident neutral atoms recombine. In this category, we also have two of the most-researched materials when it comes to γ: quartz and Pyrex. In Figure 21, the γ of glass-based materials, including quartz glass and Pyrex, is shown as determined by various researchers with respect to surface temperature. In a separate graph in Figure 22, the same results are shown with respect to pressure. As evident from Figure 21, some parallels can be drawn from different researchers as to the behavior of γ of glass-based surfaces in respect of temperature. Regrettably, the same is not true for the pressure dependence of γ Figure 22.

**Figure 23:**
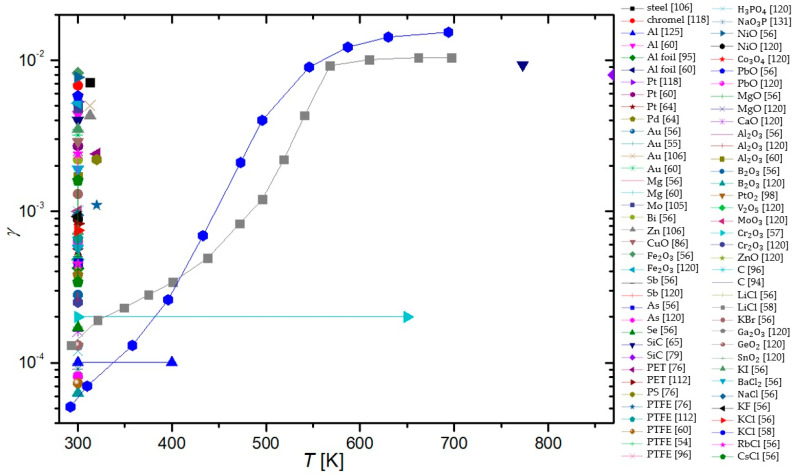
Recombination coefficient (γ) of neutral oxygen atoms versus surface temperature ($T_{s}$) for various inert surfaces.

**Figure 24:**
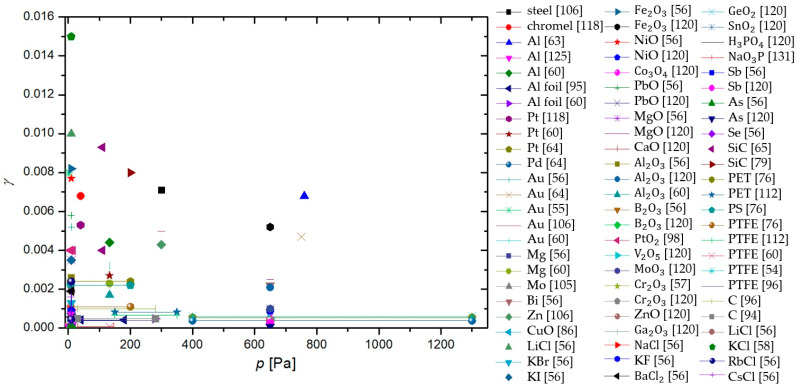
Recombination coefficient (γ) of neutral oxygen atoms versus pressure (p) for various inert surfaces.

For the rest of the inert materials, Figure 23 and Figure 24 show the evolution of *γ* versus temperature and pressure, respectively. Some common behavior of different materials can be seen: *γ* seems to increase with surface temperature. Again, nothing conclusive can be determined regarding the pressure dependence of *γ*.

## 5. Authors’ Remarks

As reported by various authors, the recombination coefficients for the same (or similar) materials differ significantly. The reasons may be due to experimental errors (which were rarely reported by the authors) but also because of other effects tackled by a few authors. They include:

- The purity of materials and their surfaces, poisoning with adsorbed gaseous species.
- The presence of other species in the gas phase and synergies between atom recombination, irradiation with photons, bombardments with positively charged ions, and surface relaxation of metastables.
- The influence of surface morphology.

The most trivial one is the purity of materials. Most materials, in particular metals, are of limited purity. The foreign atoms will enable sites for O-atom adsorption of different bunding energy than the surface atoms of the investigating materials. Furthermore, the impurity atoms may segregate on surfaces causing the formation of a very thin layer of oxide, which is chemically different from the oxides of parent materials. This effect is particularly relevant for alloys. For example, stainless steel will form various surface oxides upon exposure to oxygen plasma. The native oxide film on stainless steel is iron oxide, but the most stable is chromium oxide which forms a passive film, and this prevents further oxidation and makes stainless steel oxidation-resistant. Other oxides may be formed on the stainless steel surface as well.

**Figure 25:**
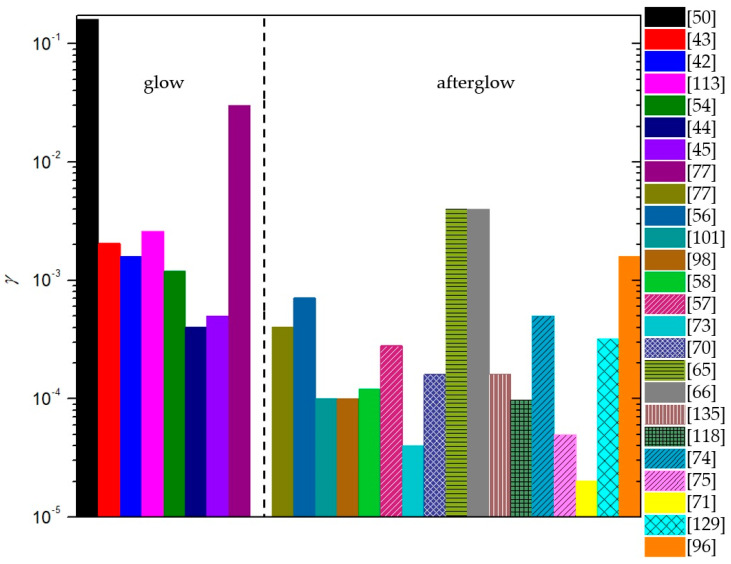
Compendium of *γ* of quartz glass measured at room temperature. Measurements in the glowing region of plasma are separated from the measurements in the afterglow.

As mentioned above, the surface recombination of O-atoms to parent molecules requires adsorption on surfaces. The number of adsorption sites is limited and governs the recombination coefficient. The number of adsorption sites depends on the chemical composition and structure of the material surface, and this property will depend on the irradiation with energetic particles. The synergy with ions (which bombard the surface with a kinetic energy that depends on the surface-to-plasma potential) and VUV radiation (which breaks bonds in the surface film of thickness equal to the penetration depth of the energetic photons) is likely to occur but was not addressed by many authors. Furthermore, the energy brought by these particles will be dissipated on the surface, and this facilitates the surface mobility of atoms. The synergy may explain the much larger coefficient measured by Cartry et al. [77] for the case treatment in glowing plasma and oxygen plasma afterglow. The kinetics of recombination versus the fluxes and/or energy of ions and photons is yet to be elaborated. The same applies to the surface relaxation of metastables and its contribution to the recombination probability. In Figure 25, measurements of γ for quartz glass (the most commonly studied material) in the plasma glow and afterglow are compared. In general, the measured γ is lower in the afterglow, although not without a few outliers.

**Figure 26:**
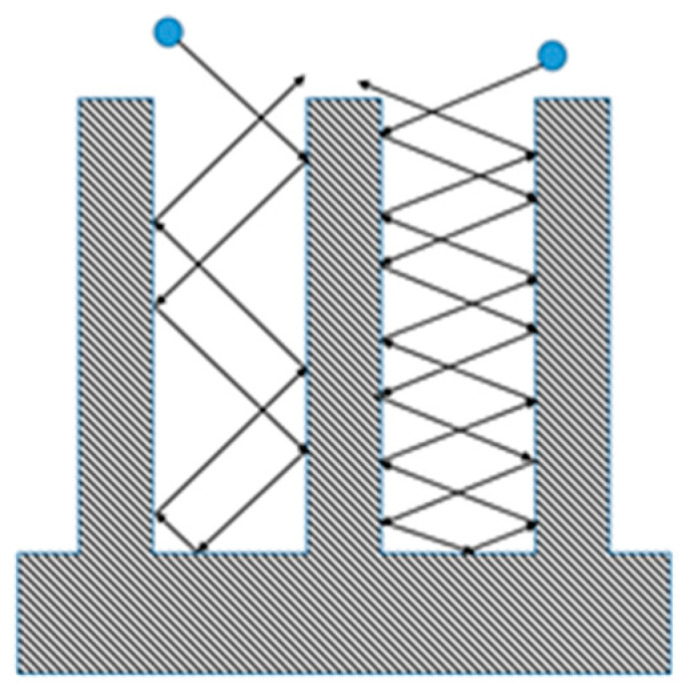
An illustration of the effect of surface morphology on the measured recombination coefficient. The arrows represent the path of atoms within the gaps in the approximation of elastic collisions with the walls.

Surface morphology was rarely reported in articles dealing with heterogeneous surface recombination of O-atoms. The effect of surface morphology on the measured recombination coefficient is evident in Figure 26. The atoms arriving from the gas phase enter the gaps in the surface of the solid material and experience numerous elastic collisions with the surface. There is a certain probability for adsorption and recombination at each collision, so the measured recombination coefficient depends on the number of collisions within the gap. In the limiting case of infinitely thin walls and infinitely long gaps, the measured recombination coefficient should approach 1, irrespective of the catalyticity of the smooth materials. The O-atoms are trapped in the material of such morphology, so they cannot leave the gap without recombining to form the parent molecules. This effect may explain the huge value of the recombination coefficient on anodized aluminum, as reported by Kurunczi et al. [124]. Namely, anodized aluminum may form a very porous structure. In fact, one of the largest coefficients was reported for carbon nanowalls [49], although amorphous carbon (or even smooth graphite) is far from being catalytic. Furthermore, the exposure of many materials to oxygen plasma at elevated temperatures may cause nanostructuring of the surface, and this increases the recombination coefficient. The influence of morphology is yet to be studied systematically.

## 6. Conclusions

In this review article, we examined various publications that reported the coefficient for heterogeneous surface recombination of oxygen atoms (γ). Special emphasis was put on finding how it changes with two key parameters: the surface temperature of the material and the pressure in the system. Knowing γ is of great importance when working with plasma since it governs our choice of materials for any given plasma-related task. If we are constructing a plasma reactor, we will search for inert materials as building blocks. On the other hand, if we want to measure plasma with a calorimetric method, we would utilize a catalytic material to increase the responsiveness of our probe. There are countless uses of oxygen plasma, and with that come countless choices of the use of proper materials.

Surface recombination may depend on the choice of material, but it still follows one of two proposed mechanisms: the Langmuir–Hinshelwood mechanism or the Eley–Rideal mechanism. The characteristic property of surface recombination is γ, which, in its purest form, is the ratio between the flux of incident neutral atoms and the flux of molecules leaving the surface. For this article, we limited ourselves to neutral oxygen surface recombinations and created a compendium of γ, determined by various authors for different materials. Experimental conditions such as the type of discharge and methods used for the evaluation of γ were also reviewed to find any possible influence on the measured γ. Discharges used by various authors can be categorized into four groups in this paper: DC, RF, MW, and other types of discharges, such as the shock tube and atomic beam discharges. γ was mostly determined using the following methods, which are also briefly explained in the paper: calorimetry, emission spectroscopy, actinometry, LIF, NO titration, Wrede–Harteck gauges, mass spectrometry, paramagnetic resonance, and numerical methods. It was found that the reported values of γ may differ by several orders of magnitude for the same material. It was difficult to draw any clear correlations because some data were not statistically significant enough. Nevertheless, the highest reported values were found for RF-ICP systems and the calorimetric method, which were the most often used by researchers.

A comparison of γ for various materials between different publications clearly demonstrates that it can be divided into three distinct categories: catalytic, semi-catalytic, and inert. For each category, we examined the dependence of γ on the surface temperature of the material and the pressure inside the experimental systems. We noticed a number of publications agreeing on the connection between surface temperature and γ. However, that was not always the case. For catalytic materials, a general increase in γ with increasing surface temperature was observed. No conclusions could be taken regarding the dependence of γ on pressure for all categories of materials. Semi-catalytic materials exhibited a variety of behaviors when examining the evolution of γ with surface temperature. However, a general rise of γ with increasing surface temperature could not be attributed to all the materials. Data on γ for inert materials was the most common across all publications due to extensive research on quartz glass and Pyrex. Inert glass materials exhibited complex behavior of γ with respect to surface temperature, while other inert materials exhibited a general rise in γ with increasing surface temperature.

This review article will serve as our basis for further research into γ of various materials, and the gathered publications as a great point of reference in further studies of surface recombinations.

## Figures and Tables

**Table 1:** Advantages and disadvantages of various types of gaseous discharges.

Plasma Source	Advantages	Disadvantages
DC	Simpler setup, lower applied voltages required	Lower atom densities, less efficient ionization
CCP RF	Reliable in sustaining plasma, scalable (widely used in industry)	Lower atom densities
ICP RF	Efficient ionization, high atom density	Induction can interfere with certain measuring methods
Pulsed RF	Less heating of plasma system	Requires quick measuring methods
MW	Efficient ionization, high atom density	Design of plasma reactor must adhere to MW wavelength
Atomic beam	Simpler setup	Source requires high temperatures, comparably lower atom densities
Shock tube	Enables study of high-energy, high-pressure discharges	Limited range of experimental conditions

**Table 2:** List of recombination coefficients (*γ*) determined with different methods for various materials in different plasma systems at a given pressure (p) and surface temperature (T). In all cases, the samples for which *γ* was determined were kept at a floating potential. Values of *γ* in parenthesis were reported for «NO-poisoned» surfaces.

Material	*γ*	p [Pa]	T [K]	Plasma	Method	Ref.
Ag	0.21	10	300	ICP RF	thermocouple probe	[56]
	0.01–0.06	40	300	atomic beam	mass spectrometry	[85]
	0.11	760	300	pulsed RF	thin film resistance thermometer + NO titration	[63]
	0.1	0.0000001	373–673	MW	silver filament thermal resistance	[108]
	0.24–0.38	1–4	310–435	ICP RF	Wrede–Harteck gauge + NO titration	[55]
	0.22–0.29	1–2	298–375	ICP RF	thermal resistivity + effusion	[99]
	0.015	300	313–423	MW	NO-titration + thermal resistivity	[106]
	0.037	400–1300	300	pulsed RF	thin-film heat transfer gauge	[64]
Ag_2_O	0.15	7–15	300	ICP RF	thin film heat-transfer gauge	[98]
	0.1	0.0000001	373–673	MW	silver filament thermal resistance	[108]
Al	0.3–0.01	1–100	400–600	ICP&CCP RF	TALIF	[50]
	0	40	300	atomic beam	mass spectrometry	[85]
	0.06–0.04	0.2–3	300	ICP RF	spinning wall desorption mass spectrometry	[126]
	0.0068	750		pulsed RF	thin film resistance thermometer + NO titration	[63]
	0.0001	1–11	300–400	ICP RF	mass spectrometry + Langmuir probe + ion flux probe	[125]
	0.05	0.2–3	310	ICP RF	Langmuir probe + actinometry + mass spectrometry	[128]
	0.0044 (0.0029)	133	300	ICP RF	NO-titration	[60]
	0.15	1–10	300	ICP RF	model	[137]
	0.4–0.6	1	300	ICP RF	spinning wall desorption mass spectrometry	[124]
Al foil	0.00042	40–180	300	ICP RF	side-arm + fiber optic catalytic probe	[95]
	0.00048	30–280	300	ICP RF	side-arm + fiber optic catalytic probe	[96]
C coated Al foil	0.0016	40–180	300	ICP RF	side-arm + fiber optic catalytic probe	[95]
Al_2_O_3_	0.0018–0.0034	10	300	ICP RF	thermocouple probe	[56]
	0.01	7–15	300	ICP RF	thin film heat-transfer gauge	[98]
	0.0021	650	300	ICP RF	Wrede–Harteck gauge	[120]
	0.014–0.031	110	313–773	pulsed RF	actinometry	[65]
	0.0097–0.061	110	300–773	pulsed RF	actinometry + NO titration	[66]
	0.085	200	940	MW	actinometry + pyrometry	[79]
	0.0017 (0.0013)	133	300	ICP RF	NO-titration	[60]
Al_2_O_3_F	0.018–0.57	200	800–2300	MW	OES + actinometry + pyrometry	[80]
Al_2_O_3_SiK	0.038–0.24	200	800–2030	MW	OES + actinometry + pyrometry	[80]
Al_2_O_3_SiK fused	0.26	200	2330–2430	MW	OES + actinometry + pyrometry	[80]
Al_2_O_3_AlN	0.0035–0.15	200	800–2000	MW	OES + actinometry + pyrometry	[80]
Al + Ni + Cr_2_O_3_	0.0036 (0.0035)	133	300	ICP RF	NO-titration	[60]
Al + PTFE	0.002 (0.002)	133	300	ICP RF	NO-titration	[60]
As	0.00046	10	300	ICP RF	thermocouple probe	[56]
	0.000081	650	300	ICP RF	Wrede–Harteck gauge	[120]
Au	0.0052	10	300	ICP RF	thermocouple probe	[56]
	0.03–0.2	40	300	atomic beam	mass spectrometry	[85]
	0.0047	750		pulsed RF	thin film resistance thermometer + NO titration	[63]
	0.008	1–4	300	ICP RF	Wrede–Harteck gauge + NO titration	[55]
	0.0061–0.012	1–2	305–347	ICP RF	thermal resistivity + effusion	[99]
	0.005	300	313	MW	NO-titration + thermal resistivity	[106]
	0.0032 (0.0019)	133	300	ICP RF	NO-titration	[60]
	0.02	400–1300	300	pulsed RF	thin-film heat transfer gauge	[64]
B_2_O_3_	0.000063	650	300	ICP RF	Wrede–Harteck gauge	[120]
	0.00028	10	300	ICP RF	thermocouple probe	[56]
BaCl_2_	0.00057–0.0019	10	300	ICP RF	thermocouple probe	[56]
BaTiO_3_	0.012–0.078	110	313–773	pulsed RF	actinometry	[65]
Bi	0.0022	650	300	ICP RF	Wrede–Harteck gauge	[120]
C	0.0005	40–180	300	ICP RF	side-arm + fiber optic catalytic probe	[94]
	0.001	30–280	300	ICP RF	side-arm + fiber optic catalytic probe	[96]
C-CAT ACC-4 SiC	0.013–0.03	500–3500	1200–1368	arc-jet	heat-flux + thermocouple + pyrometry + LIF	[104]
	0.030–0.00041	500–3500	1368–2000	arc-jet	heat-flux + thermocouple + pyrometry + LIF	[104]
CaO	0.00016	650	300	ICP RF	Wrede–Harteck gauge	[120]
CaO + ZrO_2_	0.036–0.202	200	900–2500	MW	thermocouple probe + OES + actinometry	[84]
CaTiO_3_	0.0097–0.061	110	313–773	pulsed RF	actinometry	[65]
	0.013–0.028	110	300–773	pulsed RF	actinometry + NO titration	[66]
CdO	0.001–0.27	4	295–625	ICP RF	side-arm thermocouple probe	[57]
chromel	0.0068	40	300	MW	side-arm + LIF	[118]
CNW	0.59	50	300	ICP and CCP RF	catalytic probe	[49]
Co	0.075	1–4	300	ICP RF	Wrede–Harteck gauge + NO titration	[55]
	0.14–0.08	10–400	750	ICP RF	Langmuir probe + fiber optic catalytic probe	[93]
CoO	0.029–0.034	110	300–473	pulsed RF	actinometry	[65]
Co_3_O_4_	0.0018–0.25	4	300–625	ICP RF	side-arm thermocouple probe	[57]
	0.00049	650	300	ICP RF	Wrede–Harteck gauge	[120]
constantan	0.046	40	300	MW	side-arm + LIF	[118]
Cr	0.06	1–2	308–356	ICP RF	thermal resistivity + effusion	[99]
Cr_2_O_3_	0.0002	4	300–650	ICP RF	side-arm thermocouple probe	[57]
	0.00025	650	300	ICP RF	Wrede–Harteck gauge	[120]
	0.023–0.072	300, 1000	850–1650	MW	actinometry + OES	[82]
CsCl	0.00034–0.0016	10	300	ICP RF	thermocouple probe	[56]
Cu	0.17	10	300	ICP RF	thermocouple probe	[56]
	0.07	0.02	300	ICP RF	spinning wall desorption mass spectrometry	[38]
	0.00217	25620	300	shock tube	thin film heat-transfer gauge	[40]
	0.00899	18400	300	shock tube	thin film heat-transfer gauge	[40]
	0.0213	13410	300	shock tube	thin film heat-transfer gauge	[40]
	0.016	1400	300	shock tube	thin film heat-transfer gauge	[86]
	0.4	1400	300	shock tube	thin film heat-transfer gauge	[87]
	0.031	760		pulsed RF	thin film resistance thermometer + NO titration	[63]
	0.015–0.024	1–4	300–370	ICP RF	Wrede–Harteck gauge + NO titration	[55]
	0.225	10–400	800	ICP RF	Langmuir probe + fiber optic catalytic probe	[93]
	0.070–0.11	1–2	298–375	ICP RF	thermal resistivity + effusion	[99]
	0.025	300	313	MW	NO-titration + thermal resistivity	[106]
	0.026 (0.019)	93	300	ICP RF	NO-titration	[60]
	0.23	100	55 0	ICP RF	thermocouple probe + OES + NO titration + Langmuir probe	[92]
CuO	0.0026–0.0032	14000	300	shock tube	thin film heat-transfer gauge	[86]
	0.034–0.18	4	295–485	ICP F	side-arm thermocouple probe	[57]
	0.043	650	300	ICP RF	Wrede–Harteck gauge	[120]
	0.02	10	300	ICP RF	thermocouple probe	[56]
Cu_2_O	0.1–0.34	4	300–540	ICP RF	side-arm thermocouple probe	[57]
Fe	0.036	10	300	ICP RF	thermocouple probe	[56]
	0.018	750		pulsed RF	thin film resistance thermometer + NO titration	[63]
	0.01	1–4	300	ICP RF	Wrede–Harteck gauge + NO titration	[55]
	0.41	100	550	ICP RF	2007	
Fe_2_O_3_	0.0082	10	300	ICP RF	thermocouple probe	[56]
	0.0046–0.15	4	290–625	ICP RF	side-arm thermocouple probe	[57]
	0.0052	650	300	ICP RF	Wrede–Harteck gauge	[120]
Fe_3_O_4_	0.015–0.028	110	300–873	pulsed RF	actinometry + NO titration	[66]
graphite polished	0.05	30–280	300	ICP RF	side-arm + fiber optic catalytic probe	[96]
Ga_2_O_3_	0.00013	650	300	ICP RF	Wrede–Harteck gauge	[120]
GeO_2_	0.00013	650	300	ICP RF	Wrede–Harteck gauge	[120]
H_3_PO_4_	0.00012	650	300	ICP RF	Wrede–Harteck gauge	[120]
Inconel 617 alloy	0.0014–0.18	36, 500–3500	260–1510	arc jet	side-arm + LIF	[46]
KBr	0.0013	10	300	ICP RF	thermocouple probe	[56]
KCl	0.00078	10	300	ICP RF	thermocouple probe	[56]
	0.0001–0.015	10	300–700	ICP RF	catalytic probe	[58]
KF	0.00092	10	300	ICP RF	thermocouple probe	[56]
KI	0.00074–0.0035	10	300	ICP RF	thermocouple probe	[56]
K_2_WO_3_	0.022–0.48		300		semiconductor theory (conductivity, Fermi levels)	[37]
LiCl	0.0019	10	300	ICP RF	thermocouple probe	[56]
	0.00013–0.01	10	300–700	ICP RF	catalytic probe	[58]
Li_2_WO_3_	0.022–0.052		300		semiconductor theory (conductivity, Fermi levels)	[37]
LVP SiC - SiB + ZrO_2_	0.0075–0.03	500–3500	1200–1500	arc-jet	heat-flux + thermocouple + pyrometry + LIF	[104]
	0.030–0.0016	500–3500	1500–2000	arc-jet	heat-flux + thermocouple + pyrometry + LIF	[104]
Mg	0.0026	10	300	ICP RF	thermocouple probe	[56]
	0.0023 (0.0012)	133	300	ICP RF	NO-titration	[60]
MgO	0.0035	10	300	ICP RF	thermocouple probe	[56]
	0.0089–0.022	4	295–415	ICP RF	side-arm thermocouple probe	[57]
	0.0025	650	300	ICP RF	Wrede–Harteck gauge	[120]
MgO + ZrO_2_	0.073–0.109	200	900–1600	MW	thermocouple probe + OES + actinometry	[84]
MgAl_2_O_4_ (spinel)	0.071–0.09	800	1520–1780	ICP wind tunnel PWK3	pyrometry + calorimetry + Pitot probe	[109]
	0.15	7–15	300	ICP RF	thin film heat-transfer gauge	[98]
mica	0.0012	150–350	300	double pulse DC	discharge-induced emission spectroscopy + actinometry	[112]
MnO	0.017–0.025	110	300–473	pulsed RF	actinometry	[65]
MnO_2_	0.013	650	300	ICP RF	Wrede–Harteck gauge	[120]
Mn_2_O_3_	0.0012–0.299	4	295–620	ICP RF	side-arm thermocouple probe	[57]
Mo	0.0048	10000	300	ICP RF	stagnation point heat flux + model	[105]
MoO_3_	0.02–0.007	10	300–700	ICP RF	catalytic probe	[58]
	0.001	650	300	ICP RF	Wrede–Harteck gauge	[120]
NaCl	0.00094	10	300	ICP RF	thermocouple probe	[56]
NaO_3_P	0.0000091	30–147	300	MW	electron paramagnetic resonance spectrometer	[131]
Na_2_WO_3_	0.005–0.047		300		semiconductor theory (conductivity, Fermi levels)	[37]
Nb	0.02–0.09	10–400	600	ICP RF	Langmuir probe + fiber optic catalytic probe	[93]
	0.09	100–400	420–620	ICP RF	Langmuir probe + fiber optic catalytic probe	[91]
	0.09	100	550	ICP RF	thermocouple probe + OES + NO titration + Langmuir probe	[91]
Nb nanostructured	0.8	100	550	ICP RF	thermocouple probe + OES + NO titration + Langmuir probe	[91]
Ni	0.028	10	300	ICP RF	thermocouple probe	[56]
	0.27	10–100	500–1100	ICP RF	thermocouple probe	[107]
	0.0085	750		pulsed RF	thin film resistance thermometer + NO titration	[63]
	0.017	1–4	300	ICP RF	Wrede–Harteck gauge + NO titration	[55]
	0.27	10–400	800	ICP RF	Langmuir probe + fiber optic catalytic probe	[93]
	0.27	100	550	ICP RF	thermocouple probe + OES + NO titration + Langmuir probe	[92]
	0.28	30–280	300	ICP RF	side-arm + fiber optic catalytic probe	[96]
NiO	0.0077	10	300	ICP RF	thermocouple probe	[56]
	0.04	7–15	300	ICP RF	thin film heat-transfer gauge	[98]
	0.0008–0.056	4	295–620	ICP RF	side-arm thermocouple probe	[57]
	0.1	4	300	ICP RF	NO titration	[119]
	0.00089	650	300	ICP RF	Wrede–Harteck gauge	[120]
PbO	0.0058	10	300	ICP RF	thermocouple probe	[56]
	0.00063	650	300	ICP RF	Wrede–Harteck gauge	[120]
	0.015–0.09	10	300–700	ICP RF	catalytic probe	[58]
	0.013–0.018	110	300–473	pulsed RF	actinometry	[65]
Pd	0.00038	400–1300	300	pulsed RF	thin-film heat transfer gauge	[64]
PET	0.0024	8–200	320	MW surfatron	catalytic probe	[76]
	0.00083	150–350	300	double pulse DC	discharge-induced emission spectroscopy + actinometry	[112]
PM1000	0.17–0.21	800	1500–1610	ICP wind tunnel PWK3	pyrometry + calorimetry + Pitot probe	[109]
	0.206–0.233	72–93	1499–1611	ICP wind tunnel PWK3	thermocouple + Pitot probe	[110]
	0.0003–0.16	36, 500–3500	105–1061	arc jet	side-arm + LIF	[46]
	0.046–0.19	300, 1000	850–1650	MW	actinometry + OES	[82]
	0.019–0.057	300, 1000	850–1650	MW	actinometry + OES	[82]
	0.003–0.011	300, 1000	850–1650	MW	actinometry + OES	[82]
PM 1000 sol-gel	0.003–0.49	36, 500–3500	280–1220	arc jet	side-arm + LIF	[46]
PM1000 oxidized	0.313	64	1559	ICP wind tunnel PWK3	thermocouple + Pitot probe	[110]
PP	0.02–0.3	1–100	400–600	ICP&CCP RF	TALIF	[50]
PS	0.0022	8–200	320	MW surfatron	catalytic probe	[76]
Pt	0	40	300	atomic beam	mass spectrometry	[85]
	0.014	1–4	300	ICP RF	Wrede–Harteck gauge + NO titration	[55]
	0.01–0.1	150–220	300–1100	MW	paramagnetic resonance absorption spectrometer + isothermal calorimetric detector	[73]
	0.0053	40	300	MW	side-arm + LIF	[118]
	0.0027 (0.0016)	133	300	ICP RF	NO-titration	[60]
	0.00055	400–1300	300	pulsed RF	thin-film heat transfer gauge	[64]
PtO_2_	0.004	7–15	300	ICP RF	thin film heat-transfer gauge	[98]
PTFE	0.0011	8–200	320	MW surfatron	catalytic probe	[76]
	0.00066	150–350	300	double pulse DC	discharge-induced emission spectroscopy + actinometry	[112]
	0.000073 (0.000064)	133	300	ICP RF	NO-titration	[60]
	0.0006	400, 1300	300	CCP RF	actinometry + FTIR + model	[54]
	0.0004	30–280	300	ICP RF	side-arm + fiber optic catalytic probe	[96]
Pyrex (SiO_2_ +B_2_O_3_)	0.000033–0.00012	10	300	ICP RF	thermocouple probe	[56]
	0.00019	10–100	300	ICP RF	thermocouple probe	[107]
	0.000077	10–400	300	MW	thermocouple probe	[97]
	0.0001	7–15	300	ICP RF	thin film heat-transfer gauge	[98]
	0.00013	1–4	300	ICP RF	Wrede–Harteck gauge + NO titration	[55]
	0.000031–0.000045	650	300	ICP RF	Wrede–Harteck gauge	[120]
	0.00012–0.0003	10–13	300–700	electrode + electrodeless	thermocouple probe	[58]
	0.00034–0.0011	25–660	335–410	DC glow	TALIF + actinometry + FTIR (Fourier transform IR)	[115]
	0.0004–0.016	66–626	77–460	double pulse DC	time-resolved actinometry	[47]
Pyrex	0.0039	150–350	300	double pulse DC	discharge-induced emission spectroscopy + actinometry	[112]
	0.00041–0.0052	266	300–400	DC glow	model + MW cavity + electrostatic + thermocouple probe	[102]
	0.00011–0.000061	133	300	ICP RF	NO titration	[60]
	0.0012–0.026	27; 500–3500	300–1640	arc jet	thermocouple + pyrometer + side arm	[103]
	0.000092 (0.000056)	133	300	ICP RF	NO-titration	[60]
	0.00002	26–213	300	MW magnetron	NO-titration + photometry	[72]
	0.0024	3–667	300	pulsed DC	VUV resonant absorption spectroscopy	[111]
	0.00044–0.00051	66–120	300	DC	Wrede–Harteck gauge + ozone detection	[121]
	0.006–0.05	810–2840	1435–1845	hypersonic arc jet	calorimetry + pyrometry	[134]
	0.0003–0.0036	100–1333	285–430	DC glow	time-resolved actinometry	[138]
	0.00002–0.0024	50–300	300	DC glow	actinometry + model	[113]
	0.0005	133–1330	300	MW	paramagnetic resonance	[130]
Pyrex coating	0.0058–0.0047	10000	1600–1800	ICP RF	stagnation point heat flux + model	[105]
Pyromark 2500	0.0012–0.20	36, 500–3500	465–1040	arc jet	side-arm + LIF	[46]
RbCl	0.00045–0.0024	10	300	ICP RF	thermocouple probe	[56]
RCG	0.008–0.023	400–2600	1400–1650	arc jet	stagnation point heat flux measurements + model	[100]
	0.0004–0.01	13–1330	300–1000	planetary reentry	kinetic model	[132]
Sb	0.00082	10	300	ICP RF	thermocouple probe	[56]
	0.00027	650	300	ICP RF	Wrede–Harteck gauge	[120]
Sb_2_O_3_	0.0082–0.021	110	300–473	pulsed RF	actinometry	[65]
Se	0.00017	10	300	ICP RF	thermocouple probe	[56]
Si	0.02–0.2	1–100	400–600	ICP&CCP RF	TALIF	[50]
	0.043	0.2	298–307	ICP RF	mass spectrometry + actinometry + thermal resistivity	[127]
Si + Cu	0.069	0.2	298–307	ICP RF	mass spectrometry + actinometry + thermal resistivity	[127]
SiC	0.004–0.041	110	313–773	pulsed RF	actinometry	[65]
	0.004–0.0093	110	300–773	pulsed RF	actinometry + NO titration	[66]
	0.0003–0.46	27; 500–3500	300–1320	arc jet	thermocouple + pyrometer + side arm	[103]
	0.066–0.043	800	1230–1870	ICP wind tunnel PWK3	pyrometry + calorimetry + Pitot probe	[109]
	0.085–0.009	80–902	1236–2000	ICP wind tunnel PWK3	thermocouple + Pitot probe	[110]
	0.008	200	870	MW	actinometry + pyrometry	[79]
CVD-SiC	0.003–0.03	110	300–773	pulsed RF	actinometry + NO titration	[66]
SiC oxidized	0.113–0.114	78–607	1328–1964	ICP wind tunnel PWK3	thermocouple + Pitot probe	[110]
SiC + HfBr_2_	0.052–0.0003	500–3500	1250–2000	arc-jet	heat-flux + thermocouple + pyrometry + LIF	[104]
	0.00071	10	300	ICP RF	thermocouple probe	[56]
	0.3–0.01	1–100	300	ICP&CCP RF	TALIF	[50]
	0.0001–0.0004	30	300–1200	MW	thermocouple probe inside tube	[101]
	0.000008	30	194	MW	thermocouple probe inside tube	[101]
	0.0001	7–15	300	ICP RF	thin film heat-transfer gauge	[98]
	0.00012	10	300	ICP RF	catalytic probe	[58]
	0.00028–0.0029	4	300–500	ICP RF	side-arm thermocouple probe	[57]
	0.0001–0.003	600–6600	300	DC glow	time-resolved actinometry	[42]
	0.0005	133	300	pulsed DC	VUV absorption spectroscopy + model	[45]
	0.000086–0.0004	133	300	pulsed DC	time-resolved VUV absorption spectroscopy	[44]
SiO_2_	0.003	10000	300	plasmatron MW	heat transfer + TPM (tethered particle motion) model	[78]
	0.00004	150–220	300	MW	paramagnetic resonance absorption spectrometer + isothermal calorimetric detector	[73]
	0.0004–0.01	133	300–1000	planetary reentry	kinetic model	[132]
	0.00016	650	300	ICP RF	Wrede–Harteck gauge	[120]
	0.0016–0.011	10000	570–870	ICP RF	stagnation point heat flux + model	[105]
	0.039–0.104	200	1000–1770	MW	optical pyrometry + model	[83]
	0.00016–0.014	15–16	300–900	MW	Wrede–Harteck gauge	[70]
	0.00205	13–400	300	DC glow	time-resolved modulation actinometry	[43]
	0.004–0.041	110	313–773	pulsed RF	actinometry	[65]
	0.004–0.0093	110	300–773	pulsed RF	actinometry + NO titration	[66]
	0.005–0.014	200	846–1435	MW	OES + actinometry	[81]
	0.0049–0.014	200	850–1430	MW	OES + actinometry + pyrometry	[80]
	0.00016–0.0057	266	285–1960	DC glow	model	[135]
	0.053–0.108	82–300	1180–1650	ICP wind tunnel PWK3	thermocouple + Pitot probe	[110]
	0.079–0.00039		200–400		model	[88]
SiO_2_	0.000098	40	300	MW	side-arm + LIF	[118]
	0.0005	1000–2000	300	MW	NO-titration	[74]
	0.00005	1000	300	MW	NO-titration	[75]
	0.0012	400, 1300	300	CCP RF	actinometry + FTIR + model	[54]
	0.00002	67	300	MW	NO-titration	[71]
	0.04, 0.03, 0.028	67, 133, 267	300	MW	pulsed induced fluorescence	[77]
	0.00019, 0.0004, 0.0005	67, 133, 267	300	MW	pulsed induced fluorescence	[77]
	0.033–0.029		600–1000		semiclassical collisional model	[136]
	0.0046–0.0023		600–1000		semiclassical collisional model	[136]
	0.0006–0.01	10–10000	300–1250		model	[133]
	0.0001–0.02		300–1250		model	[89]
	0.00032	100–300	300	MW	paramagnetic resonance absorption spectrometer + isothermal calorimetric detector	[129]
	0.0016	30–280	300	ICP RF	side-arm + fiber optic catalytic probe	[96]
SiO_2_ β-cristobalite	0.01–0.09	200	850–1820	MW	OES + actinometry	[81]
	0.0094–0.1	200	800–1830	MW	OES + actinometry + pyrometry	[80]
SiO_2_ + SiC	0.01	200	850	MW	actinometry + pyrometry	[79]
SiOCH nanoporous	0.0038	400, 1300	300	CCP RF	actinometry + FTIR + model	[54]
	0.004	400, 1300	300	CCP RF	actinometry + FTIR + model	[54]
	0.0044	400, 1300	300	CCP RF	actinometry + FTIR + model	[54]
SnO_2_	0.001	650	300	ICP RF	Wrede–Harteck gauge	[120]
soda glass	0.00034	10	300	ICP RF	thermocouple probe	[56]
stainless steel	0.09	1	300	pulsed RF	actinometry	[114]
	0.35–0.02	1–100	400–600	ICP&CCP RF	TALIF	[50]
	0.17	5	330	ICP RF	neutral mass spectrometry and Langmuir probe	[122]
	0.07	10–100	400–700	ICP RF	thermocouple probe	[59]
	0.04–0.16	40	300	atomic beam	mass spectrometry	[85]
	0.09	0.2–3	300	ICP RF	spinning wall desorption mass spectrometry	[61]
	0.1–0.4	2–10	300	pulsed RF	TALIF	[62]
	0.5	3	300	MW	actinometry	[69]
	0.01	40	300	CCP RF	energy resolved actinometry (fitting)	[51]
	0.013–0.005	10–400	300	CCP RF	TALIF (fitting)	[52]
	0.14	1–15	300–400	ICP RF	Langmuir probe + simulation	[123]
	0.3	1–11	300–400	ICP RF	mass spectrometry + Langmuir probe + ion flux probe	[125]
	0.09	0.2–3	310	ICP RF	Langmuir probe + actinometry + mass spectrometry	[128]
	0.5–0.1	0.1–20	300	model	model	[90]
	0.015	66	300	CCP RF	model	[53]
	0.0099 (0.0064)	133	300	ICP RF	NO-titration	[60]
	0.006–0.002	133–267	300	pulsed RF	LIF + TALIF + PIE (plasma-induced emission)	[117]
	0.07	100	550	ICP RF	thermocouple probe + OES + NO titration + Langmuir probe	[92]
	0.016	40	300	MW	side-arm + LIF	[118]
steel	0.0071	300	313	MW	NO-titration + thermal resistivity	[106]
TiO_2_	0.013–0.028	110	313–773	pulsed RF	actinometry	[65]
	0.014–0.031	110	300–773	pulsed RF	actinometry + NO titration	[66]
V_2_O_5_	0.00048	650	300	ICP RF	Wrede–Harteck gauge	[120]
W	0.013	1–4	300	ICP RF	Wrede–Harteck gauge + NO titration	[55]
WO_3_	0.017	650	300	ICP RF	Wrede–Harteck gauge	[120]
	0.012–0.078	110	313–773	pulsed RF	actinometry	[65]
W oxidized	0.035	83	1371	ICP wind tunnel PWK3	thermocouple + Pitot probe	[110]
Zn	0.0043	300	313	MW	NO-titration + thermal resistivity	[106]
ZnO	0.00034–0.1	4	295–620	ICP RF	side-arm thermocouple probe	[57]
	0.00044	650	300	ICP RF	Wrede–Harteck gauge	[120]
ZrB_2_ + 15SiC + 2MoSi_2_	0.01–0.1	200	770–1820	MW	thermocouple probe + OES + actinometry	[84]
ZrB_2_ + 15SiC + 2MoSi_2_	0.0073–0.03	200	770–2000	MW	thermocouple probe + OES + actinometry	[84]
ZrB_2_ + 10HfB_2_ + 15SiC + 2MoSi_2_	0.019–0.135	200	1000–1820	MW	thermocouple probe + OES + actinometry	[84]
ZrB_2_ + 10HfB_2_ + 15SiC + 2MoSi_2_	0.023–0.096	200	830–1820	MW	thermocouple probe + OES + actinometry	[84]
ZrO_2_ + Y_2_O_3_	0.04–0.21	200	900–2300	MW	thermocouple probe + OES + actinometry	[84]

**Table 3:** Advantages and disadvantages of various types of measuring methods for determination of the neutral atom density and recombination coefficient.

Measurement Method	Advantages	Disadvantages
Calorimetry	Simple, cheap, reliable	Slow method, slight changes of plasma conditions around detector
Emission spectroscopy	Quick, non-intrusive method	Only measurable in glow region, unreliable interpretation of data
Actinometry	Quick, non-intrusive method	Only measurable in glow region, unreliable interpretation of data
LIF	Non-intrusive method, good spatial and temporal resolution	Expensive, complicated interpretation of data
NO titration	Simple method, quick determination	Poisoning of reactor, introduction of reactive gas
Wrede–Harteck	Simple setup, only pressure detectors needed	Works only in the afterglow, needs a complimentary method
Mass spectrometry	Non-intrusive method	Complicated setup, needs simulation to interpret results
Paramagnetic resonance	Non-intrusive method	Absolute densities harder to determine, needs a complimentary method

## Data Availability

All collected and analyzed data are presented in this manuscript.
